# Fibrin clot properties in cardiovascular disease: from basic mechanisms to clinical practice

**DOI:** 10.1093/cvr/cvad017

**Published:** 2023-01-20

**Authors:** Michał Ząbczyk, Robert A S Ariëns, Anetta Undas

**Affiliations:** Thromboembolic Disorders Department, Institute of Cardiology, Jagiellonian University Medical College, 80 Pradnicka St, 31-202 Krakow, Poland; Krakow Center for Medical Research and Technologies, John Paul II Hospital, Krakow, Poland; Discovery and Translational Science Department, Leeds Institute of Cardiovascular and Metabolic Medicine, University of Leeds, Leeds, UK; Thromboembolic Disorders Department, Institute of Cardiology, Jagiellonian University Medical College, 80 Pradnicka St, 31-202 Krakow, Poland; Krakow Center for Medical Research and Technologies, John Paul II Hospital, Krakow, Poland

**Keywords:** Fibrin clot, Fibrinolysis, Cardiovascular disease, Thrombosis

## Abstract

Fibrinogen conversion into insoluble fibrin and the formation of a stable clot is the final step of the coagulation cascade. Fibrin clot porosity and its susceptibility to plasmin-mediated lysis are the key fibrin measures, describing the properties of clots prepared *ex vivo* from citrated plasma. Cardiovascular disease (CVD), referring to coronary heart disease, heart failure, stroke, and hypertension, has been shown to be associated with the formation of dense fibrin networks that are relatively resistant to lysis. Denser fibrin mesh characterized acute patients at the onset of myocardial infarction or ischaemic stroke, while hypofibrinolysis has been identified as a persistent fibrin feature in patients following thrombotic events or in those with stable coronary artery disease. Traditional cardiovascular risk factors, such as smoking, diabetes mellitus, hyperlipidaemia, obesity, and hypertension, have also been linked with unfavourably altered fibrin clot properties, while some lifestyle modifications and pharmacological treatment, in particular statins and anticoagulants, may improve fibrin structure and function. Prospective studies have suggested that prothrombotic fibrin clot phenotype can predict cardiovascular events in short- and long-term follow-ups. Mutations and splice variants of the fibrinogen molecule that have been proved to be associated with thrombophilia or increased cardiovascular risk, along with fibrinogen post-translational modifications, prothrombotic state, inflammation, platelet activation, and neutrophil extracellular traps formation, contribute also to prothrombotic fibrin clot phenotype. Moreover, about 500 clot-bound proteins have been identified within plasma fibrin clots, including fibronectin, α2-antiplasmin, factor XIII, complement component C3, and histidine-rich glycoprotein. This review summarizes the current knowledge on the mechanisms underlying unfavourable fibrin clot properties and their implications in CVD and its thrombo-embolic manifestations.

**This manuscript was handled by Reviews Deputy Editor Ali J. Marian**

## 1. Introduction

Cardiovascular disease (CVD), comprising coronary heart disease (CHD), heart failure (HF), stroke, and hypertension, remains the leading cause of mortality and hospitalization worldwide. Based on the NHANES data, the prevalence of CVD in the USA in individuals older than 20 years is 49.2% and 9.3% excluding hypertension.^1^ CVD prevalence increases with age from about 1% of the population aged 20–39 years up to 42.9% of males and 31.3% of females aged ≥80 years, excluding hypertension. The leading cause of CVD death in 2019 was CHD, accounting for 41.3% of deaths, followed by stroke in 17.2%, high blood pressure in 11.7%, HF in 9.9%, and arterial diseases in 2.8% of deaths.

Atherosclerosis underlies the vast majority of CVD.^2^ A chronic, low-grade systemic inflammation is considered as a critical factor associated with CVD with a major involvement of cellular senescence, cellular debris deposition, microbiome composition, and clonal haematopoiesis of indeterminate potential, genetic, and epigenetic components. Several studies linked increased fibrinogen levels, reflecting the effect of chronic inflammation, with increased risk of CHD and stroke [hazard ratio (HR) = 1.78, 95% confidence interval (CI) 1.19–2.66 per 1 g/L increase in plasma fibrinogen concentrations];^3,4^ however, a Mendelian randomization study revealed no causal effect of fibrinogen on CVD.^5^

**Figure 1:**
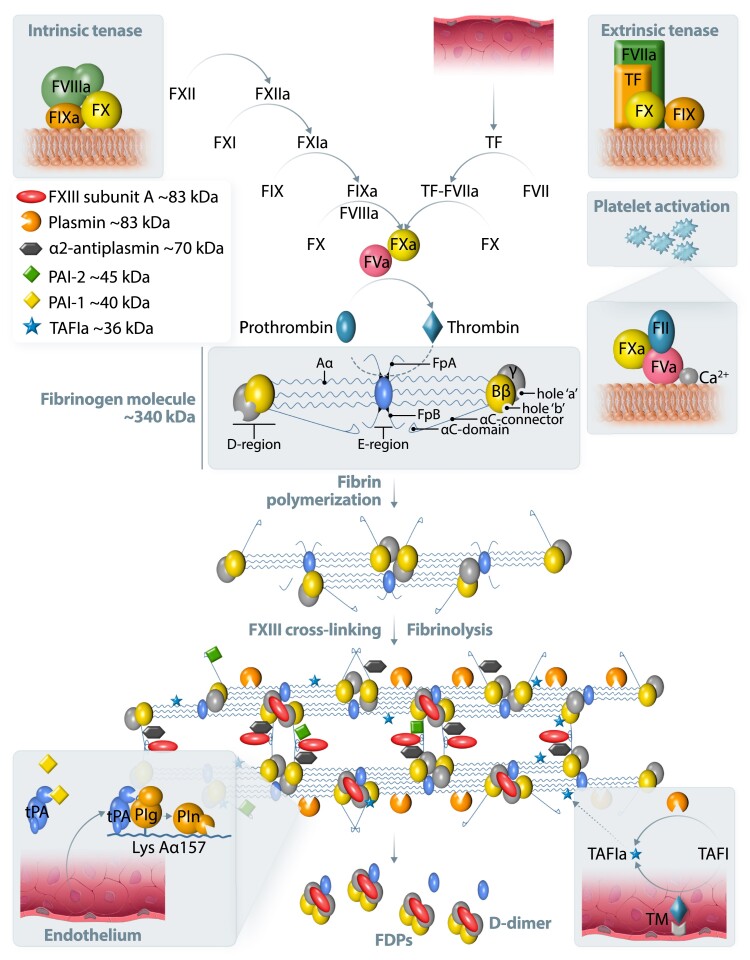
A simplified scheme of blood coagulation, fibrin formation, and fibrinolysis. Tissue factor (TF) is a trans-membrane glycoprotein present in the sub-endothelial tissue and fibroblasts. TF is not exposed to blood until the disruption of the vessel wall. TF binds to factor (F)VIIa and this complex promotes the conversion of FX to FXa (extrinsic tenase). The intrinsic pathway is induced by the activation of FXII on negatively charged surfaces followed by FXI, FIX, and finally FX activation. FIXa with its cofactor FVIIIa forms a tenase complex (intrinsic tenase), activating FX. The prothrombinase complex on activated platelets converts prothrombin (FII) to thrombin, which is a critical step in blood coagulation, preceding fibrinogen conversion to fibrin. The cleavage of fibrinopeptide A (FpA) from the fibrinogen Aα-chain exposes an N-terminal four peptide sequence, Gly-Pro-Arg-Pro (knob ‘A’). Similarly, the cleavage of FpB from the fibrinogen Bβ-chain exposes the Gly-His-Arg-Pro peptide sequence (knob ‘B’). These knobs are complementary to sequences known as hole ‘a’ and hole ‘b’ in the γ- and β-nodules of other fibrin molecules. Fibrin resistance to enzymatic degradation is limited by cross-linking by activated FXIII subunit A (*Figure 1*). Tissue- and urokinase-type plasminogen activators (tPA and uPA) convert plasminogen to plasmin. Plasminogen activator inhibitor type 1 (PAI-1) inhibits plasminogen conversion. tPA released from endothelium binds to fibrin and facilitates plasminogen binding, preferentially to lysine residues on the partially cleaved Aα chain. α2-antiplasmin, PAI-2, thrombin-activatable fibrinolysis inhibitor (TAFI) activated by a complex of thrombomodulin (TM) and thrombin inhibit fibrinolysis by inactivation of plasmin, tPA, or cleavage of lysine resides, respectively. Fibrin degradation products (FDPs) contain D-dimer as a known marker of ongoing fibrinolysis. Fibrin turnover occurs physiologically.

Conversion of circulating fibrinogen into insoluble fibrin and formation of a stable clot is the final step of the coagulation cascade, reflecting the natural ability of the organism to stop bleeding after injury. Fibrinogen, synthesized in the liver, circulates in blood at concentrations of 2–4 g/L. Human fibrinogen is a 340 kDa glycoprotein composed of three paired polypeptide chains Aα, Bβ, and γ (*Figure 1*). The six subunits are held together by 29 disulphide bonds in the central nodule of the fibrinogen molecule. Fibrinogen is composed of three main regions connected by α-helical coils, including a central E region containing the N-termini of all polypeptide chains and two outer D regions that comprise the C-termini of Bβ and γ chains (D-E-D; *Figure 1*). The C-terminal region of the Aα chain forms a globular structure located near the central E region. Thrombin specifically cleaves two fibrinopeptides A (FpA) from the N-termini of fibrinogen Aα chains, resulting in the formation of desA-fibrin monomer with exposed binding sites.^6^ The release of fibrinopeptides B (FpB) from the N-termini of the Bβ chains is not required for fibrin polymerization and takes place at a slower rate.^6^ Fibrin monomers polymerize via non-covalent interactions between the D and E regions with subsequent lateral aggregation promoted mainly by interactions of α–α chains and α–γ chains (*Figure 1*).^6^ A half-staggered fibrin forms a twisted protofibril. Lateral aggregation of double-stranded fibrin oligomers (20–25 mer protofibrils) and formation of thicker fibrin fibres is probably associated with FpB release; however, this mechanism has not been fully understood and merits further investigation. Branching is strictly required for the formation of a three-dimensional fibrin structure and a higher number of branch points is usually associated with shorter fibre segments between them.^6^ It should be noted that fibrin has a unique extensibility and single fibres can be elongated by 300–400% before rupture.^7^

Fibrin resistance to degradation by plasmin is determined mainly by covalent cross-linking, mediated by activated factor (F)XIII, which catalyses the formation of covalent bonds between γ–γ, γ–α, and α–α chains (*Figure 1*).^6^ Tissue- and urokinase-type plasminogen activators (tPA and uPA) convert plasminogen to plasmin and this process is controlled by plasminogen activator inhibitor type 1 (PAI-1). Enhanced PAI-1 release from platelets, endothelium, hepatocytes, and adipocytes directly inhibits plasminogen conversion into plasmin in circulating blood. Increased PAI-1 levels occur in CVD.^8^ In meta-analysis of the available studies, PAI-1 antigen levels, but not activity, were higher by 6.11 ng/mL (95% CI, 3.27–8.96 ng/mL) in patients with major adverse cardiac events than in controls.^9^

The catalytic efficiency of plasminogen activation by tPA, but not uPA, is greater in the presence of fibrin as a ternary complex than in the presence of fibrinogen. tPA binds to fibrin by a finger domain followed by a conformational change facilitating plasminogen binding (*Figure 1*).^10^

Increased incorporation of antifibrinolytic proteins into the fibrin mesh, such as α2-antiplasmin, PAI-2, TAFI, or complement component C3 lead to hypofibrinolysis via different mechanisms (*Figure 1*).^11^ α2-Antiplasmin and PAI-2 are cross-linked to fibrin and directly inhibit plasmin or tPA, respectively. TAFI, activated by thrombomodulin, cleaves off C-terminal lysine residues of fibrin, which are required for the binding of tPA and plasminogen.^11^

Data indicating associations between higher fibrinogen levels and increased CVD risk are inconsistent; however, fibrinogen definitely remains a valuable biomarker used in routine clinical practice.^12^ Thirty years ago it was postulated that not fibrinogen itself, but fibrin clot structure and function are involved in the development, progression, and thrombotic manifestations of CVD. This review summarizes the current knowledge on fibrin clot properties in the context of CVD and its therapy.

## 2. Assessment of fibrin clot properties

Impaired fibrin clot properties, such as fibrin network architecture, susceptibility to fibrinolysis, and biomechanical properties have been shown to be associated with a history of thrombo-embolism or recurrent events in case-control studies as well as with poor outcomes in a few large prospective trials.^13–16^ There are several laboratory assays to evaluate fibrin clot structure and function; however, most of them are not standardized and unavailable for routine laboratory assessment (*Table 1*).^17^ Plasma clotting is usually initiated using different concentrations of exogenous thrombin or tissue factor (TF), whereas in purified models thrombin is used to convert fibrinogen into fibrin.

**Table 1:** A summary of laboratory issues of standardized protocols for fibrin clot permeability (*K*_s_) and CLT assessment

Assay	Fibrin clot permeability	Common modifications	CLT	Common modifications
Sample	Citrated plasma	Plasma supplemented with RBCs or other cells	Citrated plasma	
Sample dilution	10:1	1:1, 1:4	3.33×	2–6×
Coagulation trigger	Human thrombin (1 NIH U/mL), CaCl_2_ (20 mM)	TF instead of thrombin, thrombin at a concentration of 0.1 NIH U/mL	Human thrombin (0.5 NIH U/mL), CaCl_2_ (15 mM), tPA (dose adjusted: 20–40 ng/mL to achieve CLT about 100 min), phospholipids (10 µM)	TF (0.6–6 pM) instead of thrombin; thrombin at a concentration of 0.03 NIH U/mL; tPA (for lysis assays) at a concentration of 56–83 ng/mL; lack of phospholipids
Technical aspects	Requires quick but accurate manual pipetting and much hands-on experience (2–3 s pipette mixing before putting clotting sample into the clot tube); avoiding air bubbles within the clot		Requires manual pipetting	
Temperature	Room temperature		37°C	
Equipment	Self-designed equipment	Commercially available slides for perfusion assays	96-well plate reader with a 405 nm filter	340 nm filter
Variability	3-day mean: 3.1–22% (min–max values), median, 8%	<12%	3.4–13.6% (min–max values), median, 7.7%	7–14% for lysis time
Main factors affecting results	Age, triglycerides, fibrinogen, C-reactive protein	Genetic polymorphisms, H3cit, GDF-15, antithrombin activity/antigen, HDL-C, glucose, Hcy, platelet-derived factors, use of aspirin, statin, or anticoagulants	PAI-1, TAFI, HRG	Genetic polymorphisms, sex, triglycerides, apolipoproteins, lipoprotein(a), α2-antiplasmin, fibrinogen, γ′-fibrinogen, use of aspirin, statin, or anticoagulants
Main application	Prothrombotic or bleeding tendency	VTE, CVD, metabolic syndrome/diabetes, inflammatory states, infectious diseases	Hypofibrinolysis or hyperfibrinolysis (VTE, cancer, bleeding)	CVD, metabolic syndrome/diabetes, inflammatory states, infectious diseases
Limitations	Research use Permeability range depending on the home-made equipment Hands-on experience required to obtain reproducible results Time-consuming (requires clot washing before *K*_s_ measurement)	Semi-automatization can shorten measurement time	Research use Methodological, equipment, and reagent differences contribute significantly to the outcome Data normalization necessary to compare results between laboratories	

CLT, clot lysis time; CVD, cardiovascular disease; GDF-15, growth-differentiation factor 15; H3cit, citrullinated histone H3; HDL-C, high-density lipoprotein cholesterol; HRG, histidine-rich glycoprotein; NIH, National Institutes of Health; PAI-1, plasminogen activator inhibitor type-1; TAFI, thrombin-activatable fibrinolysis inhibitor; TF, tissue factor; tPA, tissue plasminogen activator; VTE, venous thrombo-embolism.

**Figure 2:**
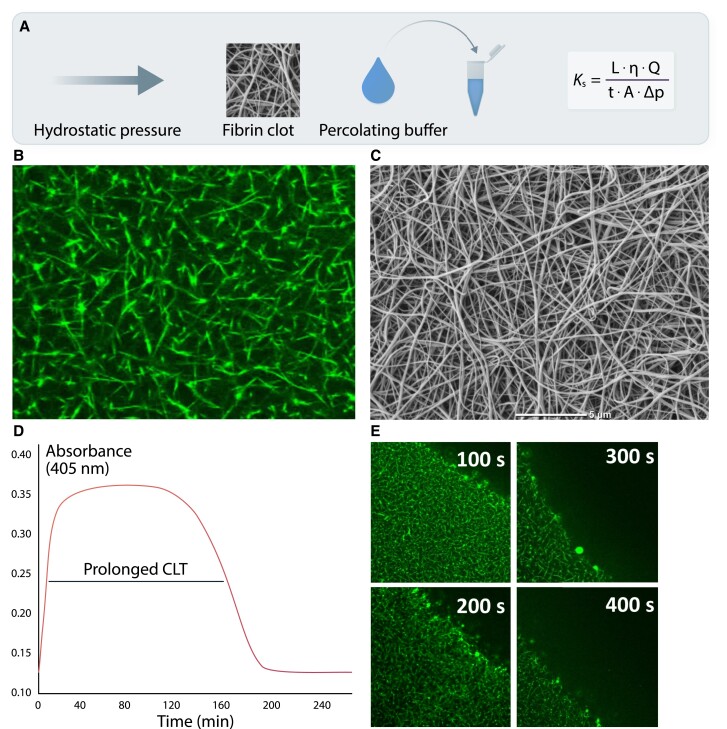
Common measures describing fibrin clot structure and function. A key measure reflecting an average pore size within a fibrin network, based on hydraulic conductivity is clot permeability or permeation (*K*_s_; *panel A*). *Q* denotes the flow rate; *L*, the length of a fibrin gel; *µ*, the viscosity of liquid (in poise); *A*, the cross-sectional area (in cm^2^), Δ*p*, a differential pressure (in dyne/cm^2^), and *t* the percolating time. Reduced *K*_s_ as a typical feature of the prothrombotic fibrin clot phenotype is usually associated with lower fibrin fibre diameter, lower pore size, and increased number of fibrin branch points visualized using confocal (*panel B*) and scanning electron microscopy (*panel C*). Faster fibrinogen polymerization results in the formation of a denser fibrin network (higher clot turbidity), which is relatively resistant to fibrinolysis as reflected by prolonged clot lysis time (CLT; *panel D*), or real-time clot lysis assessed using confocal microscopy (*panel E*).

The liquid permeability of a fibrin clot (*K*_s_ or Darcy’s constant), which reflects an average pore size within the fibrin network based on the volume of a buffer flowing through a fibrin gel in time, represents a key measure of fibrin network structure (*Figure 2A*).^18^ A lower *K*_s_ indicates a more compact and less permeable fibrin meshwork. This measure usually correlates well with a pore size assessed by confocal or scanning electron microscopy (SEM; *Figure2B* and *C*).^18^ SEM is the most commonly used method to study fibrin clot nanostructure.^19^ Investigation of the fibrin clot structure with different imaging techniques showed that the structure of hydrated clots observed using confocal microscopy is highly analogous to clot structure visualized by SEM.^20^ However, the diameter of fibrin fibres visualized using confocal microscope is sometimes 2–4 times larger than that on SEM,^21^ depending on the aggregation of fibrin monomers during protofibril formation.^22^ Frequently, there is a positive association between the mean fibrin fibre diameter on imaging and clot permeability. Denser fibrin clots are typically more resistant to lytic agents; therefore, analysis of clot permeability is supplemented by a number of lysis assays induced most commonly by varying concentrations of recombinant tPA with the use of different protocols based on turbidimetric measurements (*Figure 2D*) or real-time monitoring of changes in fibrin clot structure and movement of the lysis front on confocal microscopy (*Figure 2E*). Despite positive correlations between results of various lysis assays, they cannot be interchangeable.^23^ Functions of fibrin clot also reflect its biomechanical properties that can provide additional information on the propensity to fibrin clot to rupture and fragmentation leading to distal embolization.^15^

The subcommittee on Factor XIII and Fibrinogen of the International Society on Thrombosis and Haemostasis (ISTH) performed two international studies on the feasibility of standardized assays for assessment of *K*_s_, clot turbidity, and lysis, and concluded that both methods have a potential as future diagnostic tools.^24,25^ For example, in the clot lysis tests, several factors, such as fibrinolysis inhibitors, fibrinogen, or C-reactive protein, can affect the results, depending on the method used (type of coagulation activator, proportion of plasma, concentration of tPA, etc.).^26^ Similar observations were made for *K*_s_ measurement, where the choice of a clotting trigger can affect the results.^16^ Therefore, use of specific assays to evaluate fibrin clot properties significantly influences the presence and magnitude of differences observed in various disease states, including CVD.

It is still unknown whether plasma fibrin clot properties change over time. In apparently healthy black South Africans, clot lysis time (CLT) was prolonged by 7.3% (assay variability 3.6–4.5%) over a 10-year period and this change was determined by female gender, increasing age, obesity, increased low-density lipoprotein cholesterol (LDL-C) levels, and hyperglycaemia.^27^

Thromboelastography (TEG®; Haemonetics, Braintree, MA) and rotational thromboelastography (ROTEM®; TEM International, Munich, Germany) served as the point of care (POC) tests to assess viscoelastic properties of the whole blood clot, including clot formation and fibrinolysis in a real time and triggered by various reagents.^28^ Other POC tests, such as the Global Thrombosis Test (GTT®; Thromboquest Limited, Kent, UK) and the Total Thrombus-Formation Analysis System (T-TAS®, Fujimori Kogyo Co., Tokyo, Japan) allow for assessment of whole blood clot occlusion time under shear stress.^29^ The latter approach was also designed to evaluate thrombus formation in the presence of collagen, required for platelet activation and TF for coagulation activation.

## 3. Mechanisms of fibrin clot properties modulation

### 3.1 Proteins binding to fibrinogen/fibrin

Fibrinogen is a highly interactive (‘sticky’) protein that can bind many different binding partners. Twenty years ago, only a few proteins that bind to fibrinogen and modulate fibrin clot structure and function had been described, including decorin, platelet factor 4 (PF4), apolipoprotein(a), and fibroblast growth factor 2.^30^ More recent data strongly support the concept that the interaction of other proteins with fibrin is not a passive process and may involve the interplay of more than one binding partner. For example, FXIII catalyses the interactions of PAI-1, α2-antiplasmin, fibronectin, vitronectin, thrombospondin, collagen, and other proteins with fibrin, each of which may influence clot susceptibility for degradation and/or clot mechanical properties.^31^ About 500 clot-bound proteins have been identified within washed clots prepared from plasma obtained from healthy subjects, with the highest relative amounts for fibrinogen (about 64% of the clot mass), fibronectin (13%), α2-antiplasmin (2.7%), FXIII (1.2%), complement component C3 (1.2%), and histidine-rich glycoprotein (HRG) (0.61%).^32^ Proteins present at relative concentrations of <0.5% in the fibrin clot included (pro)thrombin, plasminogen, apolipoproteins, and PF4. A detailed analysis of fibrin clot proteome performed in patients with acute pulmonary embolism compared with healthy controls revealed higher clot-bound amounts of fibrinogen chains, apolipoprotein B-100, or histones H3 and H4 and reduced amounts of α2-antiplasmin, α2-macroglobulin, antithrombin, or plasminogen.^33^ Moreover, low *K*_s_ was associated with increased clot-bound amounts of C-reactive protein, kininogen-1, protein S, β-2-microglobulin, and thromboxane-A synthase.

Available data indicate that multiple clinical factors and laboratory parameters affect fibrin clot properties, including proteins unrelated to the coagulation system. These observations underline how complex the process of thrombosis is and how many mechanisms are engaged in its regulation. Further studies, however, are needed to evaluate to what extent particular proteins can modify fibrin clot properties and modulate the thrombotic risk.

#### 3.1.1 Thrombin

**Figure 3:**
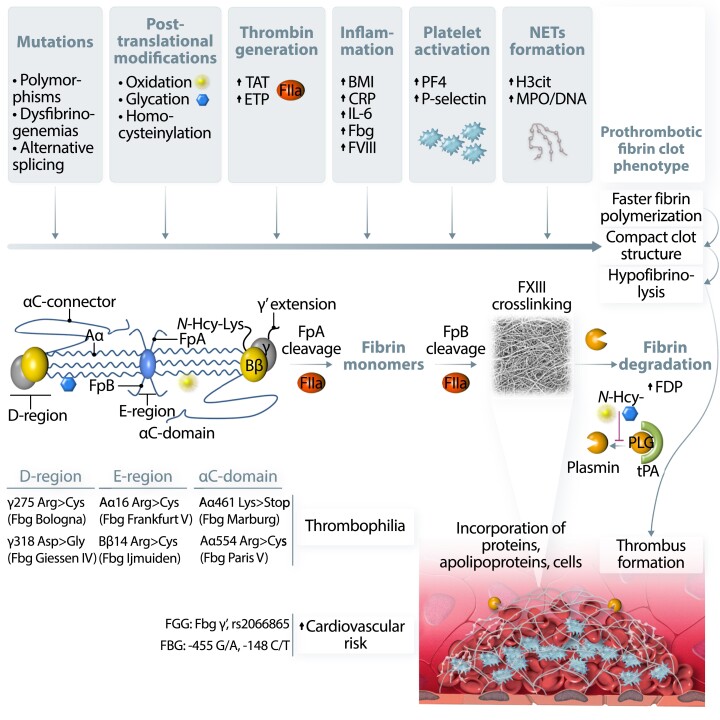
Factors and potential mechanisms involved in fibrinogen and fibrin modulation leading to prothrombotic fibrin clot phenotype. Fibrinogen (Fbg) molecule alterations, including common polymorphisms and mutations, were identified in all structural regions of this protein. Fbg is prone to post-translational modifications such as oxidation, glycation, or homocysteinylation. All these factors can influence Fbg conformation and lead to altered polymerization, resulting in the formation of dense, poorly permeable, and resistant to lysis fibrin networks, known as a prothrombotic fibrin clot phenotype. Increased prothrombin conversion to thrombin (FIIa), reflected *in vivo* by higher levels of thrombin–antithrombin (TAT) complex or *ex vivo* by increased endogenous thrombin potential (ETP) were also associated with unfavourably modified fibrin properties, probably by influencing the rate of fibrinopeptide A and B cleavage, resulting in the formation of denser fibrin clots composed of thinner fibres. Moreover, FIIa activates platelets and protein C anticoagulant systems, and inhibits fibrinolysis. Incorporation of cellular components into the fibrin network as well as binding of different proteins to fibrin by covalent and non-covalent interactions can additionally modify fibrin clot properties, especially when related to acute or chronic proinflammatory conditions. Cross-linking by factor XIII (FXIII) stabilizes fibrin structure and catalyses binding of several proteins into fibrin network. Increased body mass index (BMI) and higher levels of C-reactive protein, interleukin-6 (IL-6), or factor VIII (FVIII) were reported to be associated with prothrombotic fibrin clot phenotype in obese subjects, patients with atherosclerotic vascular disease, or individuals following venous thrombo-embolism. Release of platelet factors, such as platelet factor 4 (PF4) or P-selectin during acute thrombotic events, was also associated with the formation of dense fibrin structure resistant to enzymatic degradation. Recent data underlined an important role of neutrophil extracellular traps (NETs) formation, reflected by higher plasma levels of citrullinated histones H3 (H3cit) or myeloperoxidase (MPO)-DNA complexes in modulation of fibrin clot phenotype into more prothrombotic in acute states, such as myocardial infarction or pulmonary embolism as well as in patients with diabetes or atrial fibrillation. Faster fibrin polymerization is associated with the formation of dense and compact fibrin networks, which limits the accessibility of fibrinolytic factors. However, fibrinolysis can be limited by increased levels of plasminogen (PLG) inhibitors such as PLG activator inhibitor type 1 (PAI-1) or decreased levels of fibrinolysis activators, including tissue PLG activator (tPA). PLG conversion to plasmin is additionally inhibited by post-translational modifications of this protein, leading to a disturbed balance between coagulation and fibrinolysis during a proinflammatory state. Increased fibrin formation leads to subsequent fibrinolysis and generation of different fibrinogen degradation products (FDP), which may be incorporated into clots/thrombi altering their properties.

Thrombin is a key enzyme that converts fibrinogen into fibrin and the rate of thrombin generation during coagulation activation can determine fibrin ultrastructure (*Figure 3*).^34^ Elevated concentrations of prothrombin, which is activated by FXa in the prothrombinase complex, were associated with the formation of thinner and densely packed fibrin fibres.^35^ Therefore, medications attenuating thrombin generation (vitamin K antagonists (VKAs), heparins) or directly inhibiting thrombin (dabigatran) favourably modified fibrin clot structure and function (*Table 2*).

**Table 2:** Reported effects of therapeutic interventions on fibrin clot properties

Treatment	Study design	Effect	Reference
Aspirin	Healthy volunteers (*n* = 15) receiving aspirin (low dose, medium dose, and a single high dose)	↑↑ *K*_s_ (low dose) ↑ *K*_s_ (medium and high dose)	^36^

	Chinese hamster ovary cell lines transfected with fibrinogen grown in the absence and presence of aspirin/volunteers taking 150 mg aspirin daily for 1 week	↑ clot turbidity, ↑ *K*_s_, ↑ fibrin thickness, ↓ clot rigidity, and ↓ clot lysis	^37^
Statin	Randomized double-blind study, advanced CAD males treated with simvastatin 40 mg per day (*n* = 13) and atorvastatin 40 mg per day (*n* = 12)	↑ *K*_s_, ↑ clot maximum absorbancy, ↓ lysis time	^38^
	Prospective cohort study, 30 patients with no history of cardiovascular events assessed prior and after 3-month treatment with simvastatin (40 mg/d)	↑ *K*_s_, ↓CLT in subjects with LDL-C < 3.4 mmol/L associated with a reduction of C-reactive protein levels	^39^
	First-ever VTE patients (*n* = 28) and well-matched controls without history of VTE (*n* = 25)	↑ *K*_s_, ↑ lag phase, ↓ maximum absorbance, ↓ time to 50% lysis, ↓ CLT	^40^
	Prospective cohort study, advanced CAD patients (*n* = 130) on standard statin treatment and after 6–12 months of high-dose statin treatment (atorvastatin 80 mg/d or rosuvastatin 40 mg/d)	↑ *K*_s_, ↓ CLT in patients on the high-dose statin therapy	^41^
Blood pressure lowering therapy	Prospective cohort study, 61 patients with essential hypertension stage 1 or 2	↑ *K*_s_, ↓ lysis time, and ↓ maximal D-dimer levels released from clots	^42^
Antidiabetic therapy	The *in vitro* effect of metformin on fibrin clot properties	↓ lysis time	^43^
	The effect of insulin infusion before and after 4–6 months’ treatment of type 1 diabetes patients (*n* = 28)	↑ *K*_s_, ↑ fibre mass–length ratio	^44^
Administration of folic acid	Healthy men (*n* = 76), advanced CAD males on aspirin (*n* = 33), DM patients (*n* = 16), and patients with hypercholesterolaemia (*n* = 15)	↑ *K*_s_, ↓ lysis time in 20 asymptomatic men with hyperhomocysteinaemia	^45^
Polyunsaturated omega-3 fatty acids	Prospective, double-blind, placebo-controlled, randomized study on stable CAD patients (*n* = 54)	↑ *K*_s_, ↓ lysis time	^46^
Anticoagulants (VKAs, heparins, fondaparinux, DOACs)	*In vitro* effect of dabigatran on fibrin clot properties	↓ lysis time, ↓ clot rigidity, ↑ fibrin fibres thickness	^47^
	*In vitro* effects of apixaban, fondaparinux, and warfarin on fibrin clot porosity	↑ *K*_s_	^48^
	AF patients (*n* = 40) at the beginning of anticoagulant therapy with VKA	↑ *K*_s_, ↓ CLT after 3 days of VKA administration	^49^
	Patients following VTE before and during treatment with rivaroxaban (34 prothrombin mutation carriers and 34 non-carriers)	↑ *K*_s_, ↓ CLT after rivaroxaban intake	^50^
	Effects of rivaroxaban and apixaban on fibrinolysis using patients’ plasma, normal pooled plasma, and purified proteins	↓ lysis time	^51^
	Plasma clots of 46 acute PE patients treated with 1 mg/kg bid enoxaparin	↑ *K*_s_, ↓ CLT	^52^
	Plasma spiked with rivaroxaban, apixaban, or enoxaparin, plasma from patients on warfarin	↑ lag time, ↑ *K*_s_ depending on clotting activator	^53^

AF, atrial fibrillation; CAD, coronary artery disease; CLT, clot lysis time; DM, diabetes mellitus; DOACs, direct oral anticoagulants; *K*_s_, fibrin clot permeability; LDL-C, low-density lipoprotein cholesterol; PE, pulmonary embolism; VKA, vitamin K antagonist; VTE, venous thrombo-embolism.

#### 3.1.2 Platelet activation

Increased platelet activation has been shown to unfavourably modify fibrin clot structure and function (*Figure 3*) at least in part by releasing proteins stored in alpha-granules, such as beta-thromboglobulin or PF4 in patients with advanced atherosclerosis.^54^ P-selectin and PF4 exerted a similar effect on fibrin clot properties in diabetic patients with high cardiovascular risk.^55^ Platelet activation *per se* also resulted in the formation of stable clots, which are resistant to fibrinolysis.^56^ Platelet activation increases thrombin generation, through the assembly of tenase and prothrombinase complexes on the activated platelet membrane, which subsequently increases local fibrin clot density and stability as discussed above. Treatment with aspirin improved fibrin clot properties in stable coronary artery disease (CAD) patients,^57^ whereas aspirin withdrawal for two weeks was associated with reduced *K*_s_ by about 40% compared with values observed on treatment.^58^

Polyphosphates, linear polymers of 60–100 phosphate residues, from platelet dense granules act as FXII-driven contact pathway activators. Moreover, polyphosphates accelerate FV activation, enhance FXI activation, inhibit tissue factor pathway inhibitor, and render fibres thicker leading to poorly permeable clots.^59,60^ Polyphosphates attenuate also the binding of tPA and plasminogen to fibrin, which contributes to hypofibrinolysis.^60^

Platelets upon activation also release a2-antiplasmin, TAFI, and HRG, which negatively affect fibrin clot properties.

Importantly, platelet aggregation requires fibrinogen binding to its receptor glycoprotein IIb/IIIa (GPIIb/IIIa). Fibrin-platelet connections provide force transmission during clot contraction^61^ and, therefore, are considered as potential therapeutic targets, especially during acute thrombosis.^62,63^

#### 3.1.3 Inflammation

Chronic inflammation especially driven by elevated IL-6, as evidenced in patients with autoimmune diseases, e.g. rheumatoid arthritis or in those with chronic obstructive pulmonary disease, all considered CVD-related conditions, largely contributes to the formation of denser fibrin networks, composed of matted fibrin fibres that are more resistant to lysis compared with controls (*Figure 3*).^64–69^ C-reactive protein and IL-6 levels showed inverse associations with *K*_s_, while C-reactive protein was positively associated with time to half lysis in patients following myocardial infarction (MI).^38,70^

In septic shock patients, a complete lysis resistance of plasma clots has been noted, which was associated with reduced plasminogen activity, increased PAI-1, and lactate levels.^71^

A compact fibrin clot structure and hypofibrinolysis have been observed in patients with COVID-19.^72^ COVID-19 patients compared with patients with severe acute respiratory distress syndrome related to the influenza virus had elevated fibrinogen levels and accelerated FXII activation, which in mechanistic experiments led to the formation of compact fibrin clots composed of thin fibres with pronounced resistance to fibrinolysis, supported by higher levels of TAFI and PAI-1.^73^ Of note, acute systemic inflammation has been shown to enhance PAI-1 synthesis in adipose tissue, directly linking inflammation and hypofibrinolysis.^74^ Anti-inflammatory effects of aspirin or statins are associated with the formation of less compact fibrin clots (*Table 2*).

Neutrophil extracellular traps (NETs) are extracellular networks released from neutrophils and composed of histones, cell-free DNA, and granular enzymes (*Figure 3*). Increased concentrations of histones markedly impaired fibrinolysis *in vitro*.^75^ Clot permeability of plasma clots was reduced by about 50% in the presence of DNA or histones.^76^ The antifibrinolytic effect of DNA alone might be associated with impaired plasmin binding to fibrin due to increased affinity of plasminogen to DNA with no influence on plasmin inhibitors. Moreover, NETs retard the tPA-dependent digestion of plasma clots.^76^

NETs components have been identified within thrombi retrieved during interventional treatment from patients with acute MI (AMI), stroke, and peripheral arterial disease (PAD).^77–80^

In atrial fibrillation (AF) patients followed for a median time of 53 months, ischaemic cerebrovascular events occurred in subjects who had higher citrullinated histone H3 (H3cit) and myeloperoxidase (MPO) levels at baseline, and both *K*_s_ and CLT were weakly associated with H3cit, which explained about 5% of their variance.^81^ Increased levels of NETs markers, which were associated with enhanced inflammation, prothrombotic state, and hypofibrinolysis, were found in type 2 diabetes mellitus (DM) patients following MI compared with those without previous MI.^82^ The available data suggest that a combination of DNases with thrombolytic agents may offer a promising tool for more efficient thrombolysis at least in a subset of patients with acute thrombosis.

### 3.2 Fibrinogen alterations

#### 3.2.1 Mutations, splice variation, and polymorphisms

Congenital fibrinogen disorders include quantitative (afibrinogenaemia and hypofibrinogenaemia) and qualitative (dysfibrinogenaemia and hypodysfibrinogenaemia) abnormalities. There are several point mutations in fibrinogen Aα, Bβ, and γ chains, most commonly in the N-terminal region of the Aα chain or C-terminal region of the γ chain causing dysfibrinogenemia. In 25% of cases, dysfibrinogenemia manifests as thrombosis (*Figure 3*).^83^ It has been reported that some dysfibrinogenaemic patients with a thrombotic history have markedly abnormal fibrin clot structure and defective lysis, for example, in fibrinogen Caracas V, Dusart, and Naples.^84–86^ Dysfibrinogenaemia can lead to arterial thrombosis at a young age.^87^

Fibrinogen splice variations and polymorphisms such as *FGG* γ′ or rs2066865 and *FGB* −455 G/A or −148 C/T have been linked with an increased risk of thrombosis or CVD risk (*Figure 3*).^12^ About 12% (range 3–40%) of total plasma fibrinogen contains γ′ chain, a common fibrinogen splice variant with an additional high-affinity binding site for thrombin, circulating as γA/γ′ heterodimer or γ′/γ′ homodimer (<1% of total fibrinogen). An increase in fibrinogen γ′ may contribute to the development of CVD.^88^ It has been shown that fibrinogen γ′ directly and independently of thrombin modulates fibrin polymerization, leading to the formation of mechanically weaker clots composed of fibres with reduced protofibril packing.^89,90^ Increased γ′ fibrinogen levels have been associated with an increased risk for arterial thrombosis and stroke.^91^ Fibrinogen γ′ concentrations poorly correlated with total plasma fibrinogen in patients following MI and in population-based controls; however, increased level of γ′ fibrinogen was an independent predictor of MI [odds ratio (OR) = 1.24, 95% CI 1.01–1.52].^92^ In acute stroke patients, the ratio of fibrinogen γ′ over total fibrinogen was higher in the acute phase than 3 months after the stroke, suggesting altered fibrinogen γ mRNA processing during the acute phase.^93,94^ A genome-wide association study revealed that fibrinogen γ′ levels corresponded to CVD prevalence.^95^ In a large cohort of apparently healthy black South Africans, fibrinogen γ′ correlated stronger than total fibrinogen with CLT and was associated with CVD risk factors, such as body mass index (BMI), high-density lipoprotein cholesterol (HDL-C), metabolic syndrome, and C-reactive protein levels, explaining nearly 20% of fibrinogen γ′ variance.^96^

#### 3.2.2 Post-translational modifications

Post-translational modifications of the fibrinogen molecule such as oxidation, glycation, or homocysteinylation have been shown to affect fibrin clot structure as well as clot formation and lysis (*Figure 3*); therefore, they can contribute to thrombotic diseases.^97^

Increased production of reactive oxygen species (ROS) results in protein oxidation and the formation of carbonyl groups or amino acid modification. Fibrinogen is about 20 times more susceptible to such modifications than albumin.^97^ A variety of fibrinogen oxidative modifications performed *in vitro* (irradiation, photooxidation, ascorbate/FeCl_3_, peroxynitrite, HOCl, etc.) as well as higher levels of oxidative stress markers assessed in a few studies in patients with inflammatory and thrombotic disorders or trauma have been associated with decreased clot stiffness, reduced *K*_s_, increased clot density, and mostly with fibrin resistance to fibrinolysis.^97–101^ Among oxidative stress markers assessed in plasma, such as protein carbonyl or thiobarbituric acid reactive substances and common sources of ROS, including NADPH oxidase activity, especially fibrinogen carbonyl content correlated with altered fibrinogen polymerization and lysis in patients with systemic inflammation and in those following AMI.^100^ In subjects with acute coronary syndrome, levels of C-reactive protein and a product of lipid cell membrane peroxidation, 8-iso-prostaglandin F2α, were independent predictors of fibrin clot properties.^99^ Elevated 8-iso-prostaglandin F2α levels measured before and after coronary artery bypass graft were associated with cardiovascular and all-cause mortality.^102^ In AF patients, increased 8-isoprostane concentrations were associated with reduced *K*_s_ and thrombo-embolic events during follow-up (HR = 2.87, 95% CI 1.17–7.03), despite anticoagulant therapy.^103^

Oxidative modifications of fibrinolytic proteins, particularly of plasminogen, have also been shown to be associated with less effective fibrinolysis in patients following venous thrombosis.^104^ Data on this modification in CVD are sparse.

Glucose can non-enzymatically bind to proteins altering their function. Glycation occurs at normal blood glucose levels as a consequence of oxidative stress or at higher glucose levels in diabetic patients. Fibrinogen is prone to glycation at lysine residues^97^ and about two-fold higher fibrinogen glycation has been reported in type 2 DM patients compared with non-diabetic controls.^105^ Clots formed from fibrinogen purified from type 2 DM patients compared with controls were denser and less porous, and common clot measures, including *K*_s_, fibrin absorbance, number of branch points, and fibrin network density, correlated with HbA1c.^106^ A similar purified model showed an altered kinetics of fibrin formation resulting in reduced clot susceptibility to fibrinolysis.^107^

*N*-homocysteinylation sites on fibrinogen, associated with the formation of denser fibrin structure with reduced susceptibility to fibrinolysis, along with impaired plasminogen activation have been found on lysine residues in the fibrinogen α, β, and γ chains (α-Lys562, β-Lys344, and γ-Lys385).^108^ Elevated homocysteine levels have been reported to be associated with prothrombotic clot phenotype among CAD patients,^45^ though the relative impact of this variable in the presence of other potent modulators appears negligible in CVD.

#### 3.2.3 Fibrinogen subregions

To investigate the influence of fibrinogen subregions on fibrin formation, clot structure, and mechanics, different fibrinogen variants have been studied in the past few years. Critical functions of the αC-subregions have been shown using recombinant human fibrinogen α390 (truncated before the αC-domain) and α220 (truncated at the start of the αC-connector).^109^ Clots prepared with the α390 variant were dense and composed of thinner fibres, whereas the α220 variant was associated with the formation of porous and weak fibrin networks, which might have potential implications as therapeutic targets to reduce the risk of thrombosis. Thrombin-mediated exposure of knobs and holes on fibrin monomers during polymerization of fibrinogens with mutations in ion-pairing residues adjacent to the knob-hole site and involved in the catch-slip behaviour of fibrin bonds has shown how these residues are important for proper fibrin fibre growth and protofibril packing.^110^

A murine model with eliminated fibrin γ-chain cross-linking by FXIII formed thrombi with reduced strength, which was prone to fragmentation and increased embolization without any effect on clot size or its susceptibility to lysis.^111^

## 4. CVD risk factors

Observational studies have provided data indicating that most well-established cardiovascular risk factors are associated with prothrombotic fibrin clot properties (*Table 3*).

**Table 3:** Cardiovascular risk factors in association with fibrin clot properties

Cardiovascular risk factor	Study design	No. of subjects	Measure	Reference
Age	Case-control study	76 healthy men, 36 advanced CAD patients, 16 DM patients, 15 hypercholesterolaemic subjects	*K*_s_ correlated negatively and t50% positively with age in controls (*r* = −0.3 and *r* = 0.4), CAD (*r* = −0.65 and *r* = 0.49), and DM patients (*r* = −0.7 and *r* = 0.59, respectively; all *P* < 0.05)	^45^
	Case-control study	642 controls (and 421 MI patients)	No clear effect on CLT in controls	^112^
	Cross-sectional study	2000 healthy controls	No clear effect on CLT	^113^
	Cohort study	80 healthy controls	↑ CLT and ↑ Lys50 with increasing age	^23^
	Cohort study	2010 healthy controls	↑ clot turbidity and ↑ CLT with increasing age	^27^
Obesity	Case-control study	38 men following MI and 88 age- and sex-matched population-based controls	*K*_s_ correlated with BMI in controls (*r* = −0.27, *P* < 0.05)	^114^
	Cohort study	537 healthy subjects (plasma available for 502 individuals)	Clot maximum absorbance, AUC, and lysis time increased, whereas lysis rate decreased with an increasing number of metabolic syndrome components (all *P* < 0.0001)	^115^
	Cohort study	1288 healthy subjects, including 292 obese individuals	CLT positively associated with BMI in men (*r* = 0.42) and women (*r* = 0.37, both *P* < 0.0001)	^116^
Family history of CAD	Case-control study	100 healthy male relatives of patients with premature CAD and 100 healthy controls	↓ *K*_s_ and ↑ clot turbidity in relatives of patients	^117^
Diabetes	Case control	150 patients with type 2 diabetes and 50 controls	↓ *K*_s_ (*r* = −0.57, *P* < 0.0001), ↑ clot turbidity (*r* = 0.33, *P* < 0.0001), ↑ branch points (*r* = 0.78, *P* < 0.0001), ↑ fibrin density (*r* = 0.63, *P* < 0.0001) associated with glycated haemoglobin levels	^106^
	Interventional study	20 type 2 DM subjects	↑ *K*_s_ after achievement of glycaemic control; *K*_s_ associated with glycated haemoglobin levels at baseline (*r* = −0.63, *P* =0.038)	^107^
	Case-control	642 controls (and 421 MI patients)	No clear effect on CLT in controls	^112^
	Cohort study	211 type 2 DM patients	↓ *K*_s_, ↑ CLT, ↑ t50% in patients with DM duration >5 years compared with ≤5 years	^118^
	Case-control study	200 type 2 DM patients and 100 age- and sex-matched controls	↑ clot maximum absorbance and ↑ lysis time in DM ↓ *K*_s_ and ↑ clot maximum absorbance in DM patients with documented CVD compared with those without CVD	^119^
	Cross-sectional study	113 type 2 DM patients	*K*_s_ and CLT associated with serum levels of citrullinated H3 histone (*r* = −0.43 and *r* = 0.59, respectively) and cell-free DNA (*r* = −0.31 and *r* = 0.46, respectively; all *P* < 0.001)	^82^
Current smoking	Case-control study	642 controls (and 421 MI patients)	No clear effect on CLT	^112^
	Case control	44 male cigarette smokers and 44 non-smokers	↓ *K*_s_ and ↑ CLT	^120^
	Cross-sectional study	2000 healthy controls	No clear effect on CLT	^113^
	Case-control study	34 healthy male smokers and 34 non-smokers	↑ clot strength, ↑ clot turbidity, ↓ fibrin fibre diameter in smokers	^121^
	Cohort study	30 healthy subjects	No clear effect on CLT	^122^
Ethanol consumption	Placebo-controlled, randomized, cross-over study	18 males	↑ CLT noted after 5 h following alcohol consumption	^123^
Arterial hypertension	Cohort study	61 patients with essential arterial hypertension	↑ *K*_s_, ↓ CLT, ↓ clot resistance to lysis at 6 months of antihypertensive treatment	^42^
Hyperlipidaemia	Population-based cohort study	2010 apparently healthy subjects	↑ Fibrin maximum absorbance and ↑ CLT associated with higher LDL-C and lower HDL-C levels	^27^
	Case-control study	138 severe AS patients and 102 controls with atherosclerotic vascular disease	↑ CLT associated with total cholesterol, LDL-C, triglycerides, oxLDL, lipoprotein(a) and apolipoprotein B, C-II, C-III, and E. ↑ Lys 50 associated with apolipoprotein A-I, C-II, and C-III	^124^
	Cohort study	30 healthy subjects	LDL-C levels positively associated with CLT	^122^
	Case-control study	24 men with hyperlipidaemia following MI and 52 apparently healthy men	↓ *K*_s_, ↓ clot maximum absorbance, ↑ CLT associated with elevated plasma lipoprotein(a)	^70^

AS, aortic stenosis; AUC, area under the curve; BMI, body mass index; CAD, coronary artery disease; CLT, clot lysis time; CVD, cardiovascular disease; DM, diabetes mellitus; HDL-C, high-density lipoprotein cholesterol; *K*_s_, fibrin clot permeability; LDL-C, low-density lipoprotein cholesterol; Lys50, time to 50% lysis; MI, myocardial infarction; oxLDL, oxidized LDL.

### 4.1 Age

*K*_s_ was negatively associated with age in healthy subjects,^45^ suggesting that typical factors related to increasing age, such as low-grade proinflammatory state and impaired antioxidant ability, can alter fibrin clot structure (*Figure 3*). Interestingly, in patients with CHD or diabetes, this association was stronger than in controls.^45^ A similar effect of increasing age on prolonged lysis time has been observed in healthy controls.^23^ Other reports showed no clear association between age and fibrin clot properties;^112,113,125^ however, adjustment for demographic factors and fibrinogen levels is routinely used in most studies evaluating fibrin clot structure and function.

### 4.2 Obesity

A weak negative association of *K*_s_ with BMI was reported in healthy subjects but not in patients following MI at young age.^114^ The EuroCLOT Study has revealed that common measures of fibrin structure and function, particularly clot density and lysis time assessed by clot turbidity measurement, increased substantially with an increasing number of metabolic syndrome components, reflecting higher cardiovascular risk.^115^ In a large cohort study of 1288 healthy individuals, including 292 obese subjects, BMI and total body fat were positively associated with CLT in both men and women.^116^ Hypofibrinolysis in obese individuals is driven at least in part by increased PAI-1 levels, since adipose tissue is an additional source of PAI-1.^126^

### 4.3 Family history of CVD

Family history of CAD may also affect clot properties, suggesting an effect of still poorly defined genetic factors. Mills *et al*.^117^ have shown that male relatives of CAD patients are characterized by faster fibrin polymerization and reduced *K*_s_ compared with age-matched control subjects.

### 4.4 Diabetes

DM is a well-established cardiovascular risk factor, which in observational studies has been shown to be associated with prothrombotic fibrin clot phenotype.^106,119^ Markedly reduced *K*_s_, suggesting lower average pore size within the fibrin network, has been reported in type 2 DM patients compared with controls in a purified fibrinogen model.^106^ In plasma models, lower *K*_s_ values were mostly related to longer DM duration, poorly controlled glycaemia, and a presence of concomitant CVD.^118,119^ Patients with DM duration >5 years compared with those with the disease duration ≤5 years as well as those with HbA1c > 6.5% compared with ≤6.5% had markedly reduced *K*_s_, prolonged CLT, and time to 50% lysis.^118^ In an interventional study performed in 20 patients with type 2 DM, reduced *K*_s_ values were associated with high glycated haemoglobin (HbA1c) levels and clot porosity increased after achievement of glycaemic control.^107^

Mechanisms associated with hypofibrinolysis in type 2 DM patients have been extensively described and include enhanced thrombin generation, post-translational modifications of fibrinogen, proinflammatory state, endothelial dysfunction, and increased platelet activation.^127,128^ Enhanced incorporation of complement C3 component has also been identified as a factor linked with hypofibrinolysis in DM^129^ and a potential therapeutic target reducing the risk of thrombosis related to diabetes.^130^ Formation of NETs has been implicated in prothrombotic fibrin clot phenotype in type 2 DM patients.^82^ In CAD patients, hypofibrinolysis may help to estimate the cardiovascular risk, since the prevalence of DM increased along with increasing quartiles of lysis time.^16^ Prolonged lysis time predicted 1-year cardiovascular death (HR = 1.38, 95% CI 1.08–1.76) in diabetic patients following MI. Cardiovascular mortality in type 2 DM patients during a median follow-up of 72 months was also predicted by a higher maximum D-dimer concentration and a lower rate of D-dimer release from fibrin clots at high tPA concentration, especially in patients with a history of CVD (HR = 6.18, 95% CI 2.02–18.96 and HR = 8.98, 95% CI 2.99–26.96, respectively).^131^ Interestingly, hypoglycaemia in type 2 DM patients has also been associated with temporary and persistent prothrombotic effects, including increased density of fibrin network and hypofibrinolysis, which may influence the risk of cardiovascular mortality in diabetics.^132^

### 4.5 Cigarette smoking and alcohol intake

A few small case-control studies have shown a negative impact of cigarette smoking on *K*_s_ and clot susceptibility to lysis;^120,121^ however, in larger case-control and cross-sectional studies, the effect of smoking on CLT was not as important as other cardiovascular risk factors.^112,113^ Increased levels of oxidative stress marker 8-isoprostane were associated with impaired fibrin clot properties in smokers, suggesting that oxidation of fibrinogen and proteins involved in fibrinolysis may contribute to prothrombotic fibrin clot phenotype.

A placebo-controlled, randomized study revealed that moderate ethanol consumption, considered as a factor lowering the risk of CAD, was associated with a temporary inhibition of fibrinolysis.^123^ Ethanol intake increased PAI-1 activity; however, the exact mechanism of its increase is still unknown.

### 4.6 Arterial hypertension

Systolic blood pressure, but not diastolic pressure, has been reported to be associated with lower clot permeability and impaired clot lysability in middle-aged patients with arterial hypertension with no evidence of CVD, whereas systolic pressure reduction increased *K*_s_ and shortened lysis time at 6 months of antihypertensive treatment regardless of the class of the drugs.^42^

### 4.7 Hyperlipidaemia

Elevated LDL-C level positively correlated with clot maximum absorbance and CLT in apparently healthy individuals.^122^ In a large population-based cohort, female gender, obesity, poor glycaemic control, increased LDL-C, and decreased HDL-C were associated with clot properties progression to more prothrombotic phenotype with increasing age.^27^

Premature CAD has been associated with hyperlipidaemia along with increased fibrin fibre porosity, density, and prolonged lysis time.^133^ Intensive lowering of LDL-C levels with high-dose statins improved fibrin clot properties in subjects with no history of cardiovascular events and LDL-C below 3.4 mmol/L as well as in advanced CAD patients (*Table 3*).^38,39^

High-dose statin treatment for 6–12 months was associated with 29.2% higher *K*_s_ and CLT shortened by 16.3% in patients with a decrease in LDL-C by ≤ 1.8 mmol/L or a reduction of at least 50% if the baseline LDL-C ranged from 1.8 to 3.5 mmol/L.^41^

In severe aortic stenosis (AS), serum levels of apolipoproteins, including apolipoprotein C-III, B, A-I, and E, predicted hypofibrinolysis better than total cholesterol, LDL-C, or triglyceride levels.^124^ Of note, the association of decreased LDL-C with improvement in clot permeability and lysability supports the concept that LDL, likely via apolipoproteins, can affect the fibrin clot phenotype.

### 4.8 Increased lipoprotein(a)

Elevated levels of plasma lipoprotein(a), as a risk factor for premature CVD, have been found to be associated with reduced *K*_s_, lower clot maximum absorbance, and prolonged time to 50% lysis in healthy individuals and patients following MI, with a major contribution of small apolipoprotein(a) isoforms.^70^ Prolonged CLT in patients with severe AS was also associated with higher lipoprotein(a) levels.^124^ Lipoprotein(a) is an important carrier of oxidized phospholipids in human plasma, which contribute to atherothrombotic CVD.^134^ Moreover, lipoprotein(a) interferes with plasminogen binding sites on fibrin and, therefore, can inhibit plasminogen activation.^135^

### 4.9 Lifestyle modifications

The cardioprotective effect of physical activity is well documented and associated with reduced premature CVD morbidity.^136^ On the other hand, increased fractal dimension (df), as a marker of fibrin clot architecture and mechanical stability, has been shown in healthy individuals after extensive exercise and its values returned to baseline after 60 min resting.^137^ Higher df correlated with denser fibrin clot structure composed of smaller fibrin fibres following exercise, suggesting short-term unfavourable effects of physical activity on fibrin. However, to our knowledge, long-term effects of intense physical training on fibrin clot phenotype, especially in patients with CVD, have not been reported.

## 5. Coronary artery disease

The major cause of CVD is atherosclerosis, in which pathogenesis is closely related to chronic inflammation and lipid deposition within arterial walls, resulting in the formation of fibroatheroma and an atherosclerotic plaque. TF produced by macrophages, smooth muscle cells, and foam cells initiates the coagulation cascade in a complex with activated FVII, leading to fibrin accumulation within the atherosclerotic plaque and disease progression.^138^

Impaired fibrin clot properties are widely recognized as factors associated with poor prognosis in CAD patients and potential thrombo-embolic risk markers in this disease.^17^ However, different effects on fibrin clot structure and/or function were observed in patients with acute thrombosis or following thrombotic events. Moreover, various determinants of fibrin clot properties were identified to be associated with these conditions. The available studies on fibrin clot features performed in patients with acute and chronic CAD have been summarized in the excellent review by Kietsiriroje *et al*.^139^

### 5.1 Acute MI

The strongest evidence supporting the role of impaired fibrin clot properties in CAD is based on the report by Sumaya *et al*.^16^ The authors showed in acute coronary syndrome patients, followed for 12 months, that for each 50% increase in lysis time assessed at hospital discharge the HR for cardiovascular death adjusted for common risk factors was 1.36 (95% CI, 1.17–1.59), despite dual-antiplatelet treatment. Similar increase in maximum turbidity, a measure of fibrin clot density, was associated with cardiovascular death (HR = 1.24, 95% CI 1.03–1.50), but this association was not significant after adjustment for biomarkers such as C-reactive protein, troponin T, and N-terminal pro B-type natriuretic peptide (NT-proBNP). Lysis time added to a clinical predictive model, including randomization to clopidogrel or ticagrelor, age, gender, BMI, smoking history, hypertension, dyslipidaemia, DM, chronic kidney disease, ST-elevation MI and previous MI, congestive heart failure, revascularization, ischaemic stroke, or PAD, displayed an incremental prognostic value for cardiovascular death (C-index 0.7 [0.649–0.75] vs. 0.69 [0.642–0.741], *P* < 0.001).^16^ Impaired clot lysability predicted also 1-year cardiovascular death (HR = 1.38; 95% CI 1.08–1.76 for each 50% increase in lysis time) and cardiovascular death combined with MI (HR = 1.21; 95% CI 1.02–1.44 for each 50% increase in lysis time) in DM patients following acute coronary syndrome.^140^

**Figure 4:**
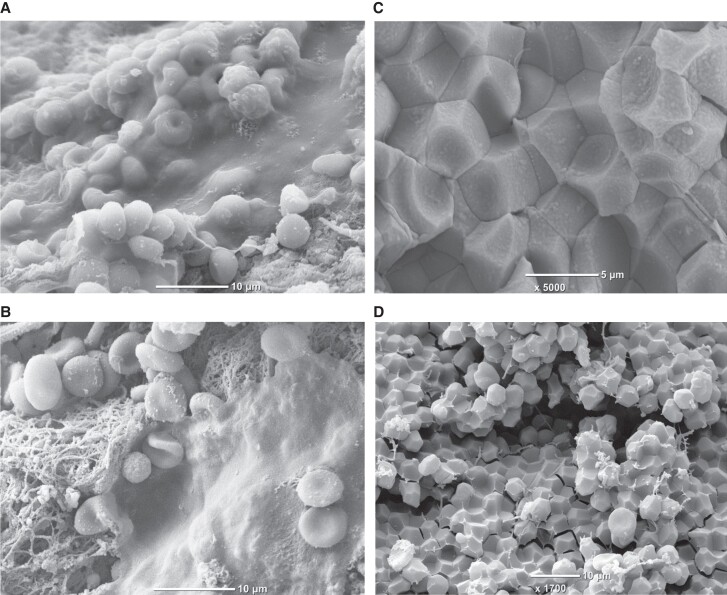
Fibrin biofilm covering intracoronary thrombi retrieved from acute myocardial infarction patients during thrombectomy (*panel A and B*) and polyhedrocytes formed *in vivo* within intracoronary thrombus (*panel C*) or *ex vivo* within whole blood clot (*panel D*). Images from ref.^143^ (*panel A and B*) are reproduced with permission from the Editorial Office of *Cardiovascular Research*.

Reduced *K*_s_, faster fibrin polymerization, and prolonged lysis time, associated with oxidative stress and the extent of inflammation, were reported in AMI patients compared with well-matched controls with stable angina.^99^ Prolonged lysis time (>90th percentile value in the control group) has been reported as a risk factor for MI (crude OR = 3.08, 95% CI 2.27–4.19).^141^ Prothrombotic fibrin clot features in AMI were shown to be associated with increased thrombin generation and platelet activation markers.^54^ Moreover, high fibrin content within the intracoronary thrombi obtained during thrombectomy correlated with low *K*_s_^142^ and higher plasma levels of platelet markers.^54^ Formation of a thin fibrin film on the surface of some intracoronary thrombi, retrieved during thrombectomy from AMI patients, suggested that additional mechanisms may limit thrombus degradation and fragmentation *in vivo* (*Figure 4A*).^143^ The role of fibrin biofilm in thrombosis is not known so far; however, a murine model showed its protective function against microorganisms during the wound healing process.^144^

Another possible mechanism that limits thrombus dissolution is its retraction. Contractile forces are generated by activated platelets interacting with fibrin, which change the shape of internally packed red blood cells (RBCs), forming polyhedral structures called polyhedrocytes (*Figure 4B*). Polyhedrocytes have been identified within arterial (*Figure 4C*) and venous thrombi,^145,146^ and it has been suggested that reduced clot contraction can contribute to thrombo-embolic complications.^147,148^ Of note, RBCs incorporation into plasma fibrin clots resulted in heterogeneous clot structure, increased fibre diameter, and increased viscoelastic moduli with no influence on clot permeability.^149^ The presence of RBCs also increased the stiffness of clots prepared from the γ′ fibrinogen.^150^

In a recent study, less porous clots with densely packed fibres and decreased number of protofibrils were observed in AMI patients compared with healthy controls.^151^ Available data show that patients with acute coronary syndrome are characterized by formation of dense fibrin clots, prolonged CLT or time to 50% lysis, and formation of stiffer fibrin clots. Such prothrombotic clot features were shown to be associated with the inflammatory state and oxidative stress, which are known to be implicated in atherosclerosis development and progression as well as with increased thrombin generation and platelet activation observed in thrombotic complications. In contrast to stable CAD, which is mostly associated with hypofibrinolysis, increased fibrinogen levels observed in acute coronary syndromes may additionally alter fibrin clot structure, resulting in the formation of dense fibrin clots. Fibrinogen level explains about 18% of variations in *K*_s_^152^ and 0.5% of the variance in CLT.^113^ Dense fibrin network may reduce accessibility of fibrinolytic factors within fibrin mesh, but clot susceptibility to lysis is mostly limited by the level and activity of profibrinolytic factors and fibrinolysis inhibitors.

### 5.2 Stable CAD or a history of MI

Reduced *K*_s_ characterizes advanced CAD patients compared with healthy controls.^45,114^*K*_s_ values reported in stable CAD were higher than those observed in patients with acute coronary syndrome.^99^

Hypofibrinolysis defined as CLT in the fourth quartile compared with the first quartile has been identified as a risk factor for MI in men aged <50 years (OR 3.2, 95% CI 1.5–6.7).^112^ Stable CAD patients were characterized by enhanced fibrin formation and impaired fibrinolysis associated with PAI-1 levels.^153^ A cross-sectional study has shown that among individuals without documented CVD, women with coronary plaques had impaired clot susceptibility to lysis compared with subjects without coronary plaques.^154^ Moreover, increased area under the curve of clot formation and lysis predicted cardiovascular events (HR = 2.4, 95% CI 1.2–4.6) in stable CAD patients.^155^ A population-based case-control study confirmed that prolonged CLT was associated with an increased risk of MI (OR = 2.8, 95% CI 1.7–4.7).^156^ Cardiovascular and all-cause deaths were associated with prolonged CLT (HR per 10 min 1.206, 95% CI 1.037–1.402; and HR 1.164, 96% CI 1.032–1.309, respectively).^157^

Concomitant type 2 DM in CAD patients has been identified as a factor associated with impaired fibrin clot structure and hypofibrinolysis,^158,159^ suggesting that type 2 DM is potent enough to affect fibrin clot properties in CAD. The AB0 risk allele rs495828 was also associated with the formation of a more compact fibrin network in stable CAD.^38,160^ On the other hand, statins, fibrates, and angiotensin-converting enzyme inhibitors have been shown to improve fibrin density and its susceptibility to lysis in CAD patients.^38,41,161^ However, it remains to be established whether any specific modulators of fibrinolytic efficiency might be useful in the prevention of clinical outcomes in atherosclerotic vascular disease.

## 6. Heart failure

Prothrombotic fibrin clot phenotype, including faster fibrin polymerization, reduced *K*_s_, lower clot compaction, and a trend to prolonged lysis time have been reported in patients with chronic HF with sinus rhythm compared with well-matched controls.^162^ C-reactive protein, fibrinogen, and thrombin–antithrombin complex levels had a major contribution to fibrin clot properties in chronic HF.^162^ Interestingly, left atrium diameter was positively correlated with lysis time.^162^ It has also been shown that patients with acute HF compared with both chronic HF and CAD patients (all taking aspirin) were characterized by higher rate of clot formation, higher clot density, slower rate of clot dissolution, and prolonged time to 50% clot lysis.^163^ Since the common causes of HF are conditions known to be associated with prothrombotic fibrin clot phenotype, such as CAD, hypertension, or AF, the mechanism of fibrin properties alteration in HF is complex and mostly related to enhanced inflammation and thrombin generation.

## 7. Stroke

In contrast to CAD patients, data on fibrin clot features in patients with acute stroke are limited due to the heterogeneity of stroke pathophysiology, subtypes, and various treatment strategies. Patients with acute ischaemic stroke assessed within 72 h of symptoms onset were characterized by reduced *K*_s_ and prolonged lysis time compared with healthy controls.^164^ Formation of denser fibrin networks resistant to lysis was observed in patients with acute ischaemic stroke before thrombolytic therapy, and prolonged CLT assessed at baseline predicted adverse neurological outcomes at 3 months.^165^

The composition of thrombi retrieved from stroke patients is highly heterogeneous, and similarly to intracoronary thrombi, these thrombi contained mostly fibrin, platelets, and RBCs.^166^ Fibrin-rich clots compared with RBC-rich clots were associated with an increased number of recanalization manoeuvres, longer time of thrombectomy, and worse clinical outcomes.^166^ Since the fibrin amount within intracoronary thrombi correlated with plasma-derived *K*_s_ in AMI patients,^142^ a similar association can be expected in ischaemic stroke patients. However, further studies are needed to confirm this hypothesis. Prothrombotic fibrin clot features in acute ischaemic stroke patients were associated with increased thrombin generation and higher levels of fibrinogen or lipoprotein(a),^164,165^ suggesting that multiple factors are involved in altering clot properties in this acute state.

On the other hand, young patients following acute cerebral ischaemia were characterized by enhanced thrombin generation, platelet activation, and hypofibrinolysis, despite the use of antithrombotic treatment in 75% of the studied subjects.^167^ Ischaemic stroke patients assessed 3–19 months after the event compared with healthy controls had reduced clot permeability, faster fibrin polymerization, and prolonged CLT, despite similar fibrinogen levels.^168^ SEM analysis revealed increased fibrin diameter and density in cryptogenic stroke patients.^168^ Siegerink *et al*.^156^ reported that hyperfibrinolysis, defined as the first tertile of CLT in the control group, was associated with about four-fold increased risk of ischaemic stroke among patients assessed at least 23 months after the event. The available data suggest a tendency to hypofibrinolysis, at least in a subset of patients following ischaemic stroke.

## 8. Peripheral arterial disease

Impaired fibrin clot properties, largely driven by hypofibrinolysis, were observed in patients with intermittent claudication, a manifestation of PAD associated with about six-fold higher risk of cardiovascular mortality.^169^ First-degree relatives of intermittent claudication patients were characterized by thicker fibrin fibres measured by turbidity, increased factor FXIII cross-linking activity, and resistance to fibrinolysis.^170^

Hypofibrinolysis, along with a significant reduction in *K*_s_, was reported in 106 PAD patients compared with controls.^171^ Both fibrin measures predicted PAD progression during long-term follow-up.^171^ A history of acute limb ischaemia (ALI), the most serious PAD presentation, was associated with reduced *K*_s_ and decreased rate of D-dimer release from clots.^172^ Premature PAD has also been associated with reduced *K*_s_ and prolonged lysis time compared with the control group without PAD.^173^ Restenosis in ALI patients, detected during one-year follow-up was associated with prothrombotic fibrin features at baseline, along with increased thrombin generation and higher von Willebrand factor antigen levels.^174^ Despite similar risk factors for CAD and PAD, impaired fibrin clot structure, especially clot porosity reflected by low *K*_s_, seems to be characteristic of the latter disease. Impaired fibrin clot structure can be driven by elevated fibrinogen levels.^152^ However, taking the intricate interactions between atherosclerosis, inflammation, and coagulation activation in PAD patients into account, it is hard to determine specific factors associated with impaired fibrin clot properties in this disease.

## 9. Arrhythmia

AF is the most common cardiac arrhythmia, affecting up to one-quarter of the adult population. AF is associated with an increased risk of stroke or systemic embolism. Currently, there is a need for biomarkers to assess both thrombotic and bleeding risk in AF,^175^ especially in subjects with additional risk factors, such as chronic kidney disease.^176,177^ Major bleeding is the most feared adverse event in anticoagulated AF patients.^178^ The prevalence of major bleeding in AF is even higher than the incidence of stroke or systemic embolism on non-VKA oral anticoagulants (NOACs; 2–4% vs. 1.5–2.5%). In AF patients at the onset of oral anticoagulation, plasma fibrin clot properties were improved after 3 days of VKA administration,^49^ whereas treatment with rivaroxaban was associated with markedly increased *K*_s_ and shortened CLT 2–6 h after its intake.^50^

Formation of compact plasma fibrin clots that are more resistant to lysis has been observed in AF patients, regardless of the AF type and CHA_2_DS_2_-VASc or HAS-BLED scores.^179,180^ Reduced *K*_s_ was associated with increased risk of both ischaemic stroke/transient ischaemic attack (HR 6.55; 95% CI 2.17–19.82) and major bleeds (HR 10.65; 95% CI 3.52–32.22) in AF patients treated with VKA.^181^ Lower *K*_s_ also predicted ischaemic cerebrovascular events (HR 6.64; 95% CI 2.2–20.1) and major bleeding events (HR 7.38; 95% CI 2.58–21.10) in AF patients taking rivaroxaban.^182^ Long-term follow-up studies on large populations are, however, needed to confirm the clinical utility of the fibrin clot phenotype assessment in AF.

## 10. Aortic aneurysm

Unfavourably altered fibrin clot properties were identified in patients with aortic aneurysm, which aetiology is under investigation, but includes trauma, infection, and inflammation.

It has been shown that patients with abdominal aortic aneurysms form dense fibrin clots with smaller pores, which are resistant to fibrinolysis.^183^ Both clot porosity, reflected by *K*_s_, and time to 50% lysis of the clot were associated with the size of aneurysm. Elevated plasma thrombin–antithrombin complex levels along with increased D-dimer concentrations independently predicted the growth of abdominal aortic aneurysms.^184^ These observations suggest an important involvement of coagulation activation and fibrin formation in the disease progression and a potential link between abdominal aortic aneurysm and coronary atherosclerosis, which share common risk factors. Therefore, it has been hypothesized that patients with abdominal aortic aneurysm may benefit from an early evaluation of coronary arteries.^185^

Assessment of fibrin clot properties in patients with isolated abdominal aortic aneurysms might help to clarify to what extent fibrin measures can predict unfavourable clinical outcomes in such subjects.

## 11. Heart valve disease

There is evidence for the prothrombotic clot phenotype in patients with severe AS free of CAD and PAD. Systemic hypofibrinolysis, reflected by prolonged CLT, was associated with the severity of AS as well as larger amounts of valvular fibrin and higher PAI-1 valvular expression.^186^ Even more pronounced prolongation of lysis time was observed in patients with degenerative mitral valve stenosis compared with AS.^187^ Increased oxidative stress and elevated levels of apolipoprotein C-III, B, A-I, and E have been implicated in prothrombotic fibrin clot phenotype in severe AS patients.^124,188^ Interestingly, high-risk AS patients scheduled for transcatheter aortic valve implantation (TAVI) compared with those undergoing surgical aortic valve replacement formed denser and more resistant to lysis fibrin clots.^189^ After adjustment for age and clinical risk factors, prolonged lysis time was an independent predictor for TAVI indication. Clinical risk factors known to alter fibrin clot properties such as concomitant DM can additionally impair fibrin clot phenotype in AS patients.^190^ Therapeutic interventions that inhibit valvular inflammation and coagulation activation may slow the rate of AS progression, whereas plasma clot susceptibility to fibrinolysis might be a useful marker reflecting the risk of thrombotic complications in AS.

## 12. Concluding remarks

Impaired plasma fibrin clot properties, largely determined by environmental factors, characterize patients with acute and chronic manifestations of CVD and less favourable features may identify subjects at high risk of recurrent thrombo-embolic events as well as faster progression of CVD. Given challenges of measurement standardization in practice, clinical implications of clot permeability or other clot fibrin measures remain to be established. Clarification of multiple mechanisms underlying the formation of more compact fibrin networks might help develop novel therapies aimed at favourable modification of clot structure.

## Authors’ contribution

M.Z. reviewed the literature and drafted the manuscript, and R.A. and A.U. revised and edited the manuscript.

## Data Availability

No new data were generated or analysed in support of this research.

## References

[1] Tsao CW , Aday AW, Almarzooq ZI, Alonso A, Beaton AZ, Bittencourt MS, Boehme AK, Buxton AE, Carson AP, Commodore-Mensah Y, Elkind MSV, Evenson KR, Eze-Nliam C, Ferguson JF, Generoso G, Ho JE, Kalani R, Khan SS, Kissela BM, Knutson KL, Levine DA, Lewis TT, Liu J, Loop MS, Ma J, Mussolino ME, Navaneethan SD, Perak AM, Poudel R, Rezk-Hanna M, Roth GA, Schroeder EB, Shah SH, Thacker EL, VanWagner LB, Virani SS, Voecks JH, Wang N-Y, Yaffe K, Martin SS. Heart disease and stroke statistics-2022 update: a report from the American Heart Association. Circulation 2022;145:e153–e639. 35078371 10.1161/CIR.0000000000001052

[2] Liberale L , Badimon L, Montecucco F, Lüscher TF, Libby P, Camici GG. Inflammation, aging, and cardiovascular disease: JACC review topic of the week. J Am Coll Cardiol 2022;79:837–847. 35210039 10.1016/j.jacc.2021.12.017 PMC8881676

[3] ten Cate H , Meade T. The Northwick Park Heart Study: evidence from the laboratory. J Thromb Haemost 2014;12:587–592. 24593861 10.1111/jth.12545

[4] Fibrinogen Studies Collaboration, Danesh J, Lewington S, Thompson SG, Lowe GDO, Collins R, Kostis JB, Wilson AC, Folsom AR, Wu K, Benderly M, Goldbourt U, Willeit J, Kiechl S, Yarnell JWG, Sweetnam PM, Elwood PC, Cushman M, Psaty BM, Tracy RP, Tybjaerg-Hansen A, Haverkate F, de Maat MPM, Fowkes FGR, Lee AJ, Smith FB, Salomaa V, Harald K, Rasi R, Vahtera E, Jousilahti P, Pekkanen J, D’Agostino R, Kannel WB, Wilson PWF, Tofler G, Arocha-Piñango CL, Rodriguez-Larralde A, Nagy E, Mijares M, Espinosa R, Rodriquez-Roa E, Ryder E, Diez-Ewald MP, Campos G, Fernandez V, Torres E, Marchioli R, Valagussa F, Rosengren A, Wilhelmsen L, Lappas G, Eriksson H, Cremer P, Nagel D, Curb JD, Rodriguez B, Yano K, Salonen JT, Nyyssönen K, Tuomainen T-P, Hedblad B, Lind P, Loewel H, Koenig W, Meade TW, Cooper JA, de Stavola B, Knottenbelt C, Miller GJ, Cooper JA, Bauer KA, Rosenberg RD, Sato S, Kitamura A, Naito Y, Palosuo T, Ducimetiere P, Amouyel P, Arveiler D, Evans AE, Ferrieres J, Juhan-Vague I, Bingham A, Schulte H, Assmann G, Cantin B, Lamarche B, Després J-P, Dagenais GR, Tunstall-Pedoe H, Woodward M, Ben-Shlomo Y, Davey Smith G, Palmieri V, Yeh JL, Rudnicka A, Ridker P, Rodeghiero F, Tosetto A, Shepherd J, Ford I, Robertson M, Brunner E, Shipley M, Feskens EJM, Kromhout D, Dickinson A, Ireland B, Juzwishin K, Kaptoge S, Lewington S, Memon A, Sarwar N, Walker M, Wheeler J, White I, Wood A. Plasma fibrinogen level and the risk of major cardiovascular diseases and nonvascular mortality: an individual participant meta-analysis. JAMA 2005;294:1799–1809. 16219884 10.1001/jama.294.14.1799

[5] Ward-Caviness CK , de Vries PS, Wiggins KL, Huffman JE, Yanek LR, Bielak LF, Giulianini F, Guo X, Kleber ME, Kacprowski T, Groß S, Petersman A, Davey Smith G, Hartwig FP, Bowden J, Hemani G, Müller-Nuraysid M, Strauch K, Koenig W, Waldenberger M, Meitinger T, Pankratz N, Boerwinkle E, Tang W, Fu Y-P, Johnson AD, Song C, de Maat MPM, Uitterlinden AG, Franco OH, Brody JA, McKnight B, Chen Y-DI, Psaty BM, Mathias RA, Becker DM, Peyser PA, Smith JA, Bielinski SJ, Ridker PM, Taylor KD, Yao J, Tracy R, Delgado G, Trompet S, Sattar N, Jukema JW, Becker LC, Kardia SLR, Rotter JI, März W, Dörr M, Chasman DI, Dehghan A, O’Donnell CJ, Smith NL, Peters A, Morrison AC. Mendelian randomization evaluation of causal effects of fibrinogen on incident coronary heart disease. PLoS One 2019;14:e0216222. 31075152 10.1371/journal.pone.0216222 PMC6510421

[6] Litvinov RI , Pieters M, de Lange-Loots Z, Weisel JW. Fibrinogen and fibrin. Subcell Biochem 2021;96:471–501. 33252741 10.1007/978-3-030-58971-4_15

[7] Liu W , Jawerth LM, Sparks EA, Falvo MR, Hantgan RR, Superfine R, Lord ST, Guthold M. Fibrin fibers have extraordinary extensibility and elasticity. Science 2006;313:634. 16888133 10.1126/science.1127317 PMC1950267

[8] Pavlov M , Ćelap I. Plasminogen activator inhibitor 1 in acute coronary syndromes. Clin Chim Acta 2019;491:52–58. 30659821 10.1016/j.cca.2019.01.013

[9] Jung RG , Motazedian P, Ramirez FD, Simard T, di Santo P, Visintini S, Faraz MA, Labinaz A, Jung Y, Hibbert B. Association between plasminogen activator inhibitor-1 and cardiovascular events: a systematic review and meta-analysis. Thromb J 2018;16:12. 29991926 10.1186/s12959-018-0166-4 PMC5987541

[10] Kim PY , Tieu LD, Stafford AR, Fredenburgh JC, Weitz JI. A high affinity interaction of plasminogen with fibrin is not essential for efficient activation by tissue-type plasminogen activator. J Biol Chem 2012;287:4652–4661. 22187433 10.1074/jbc.M111.317719 PMC3281636

[11] Pechlivani N , Kearney KJ, Ajjan RA. Fibrinogen and antifibrinolytic proteins: interactions and future therapeutics. Int J Mol Sci 2021;22:12537. 34830419 10.3390/ijms222212537 PMC8625824

[12] Surma S , Banach M. Fibrinogen and atherosclerotic cardiovascular diseases—review of the literature and clinical studies. Int J Mol Sci 2021;23:193. 35008616 10.3390/ijms23010193 PMC8745133

[13] Undas A , Ariëns RAS. Fibrin clot structure and function: a role in the pathophysiology of arterial and venous thromboembolic diseases. Arterioscler Thromb Vasc Biol 2011;31:e88–e99. 21836064 10.1161/ATVBAHA.111.230631

[14] Ząbczyk M , Undas A. Plasma fibrin clot structure and thromboembolism: clinical implications. Pol Arch Intern Med 2017;127:873–881. 29225327 10.20452/pamw.4165

[15] Feller T , Connell SDA, Ariёns RAS. Why fibrin biomechanical properties matter for hemostasis and thrombosis. J Thromb Haemost 2022;20:6–16. 34528378 10.1111/jth.15531

[16] Sumaya W , Wallentin L, James SK, Siegbahn A, Gabrysch K, Bertilsson M, Himmelmann A, Ajjan RA, Storey RF. Fibrin clot properties independently predict adverse clinical outcome following acute coronary syndrome: a PLATO substudy. Eur Heart J 2018;39:1078–1085. 29390064 10.1093/eurheartj/ehy013 PMC6019045

[17] Larsen JB , Hvas A-M. Fibrin clot properties in coronary artery disease: new determinants and prognostic markers. Pol Arch Intern Med 2021;131:16113. 34623063 10.20452/pamw.16113

[18] Undas A . How to assess fibrinogen levels and fibrin clot properties in clinical practice? Semin Thromb Hemost 2016;42:381–388. 27071050 10.1055/s-0036-1579636

[19] Veklich Y , Francis CW, White J, Weisel JW. Structural studies of fibrinolysis by electron microscopy. Blood 1998;92:4721–4729. 9845538

[20] Hethershaw EL , Cilia La Corte AL, Duval C, Ali M, Grant PJ, Ariëns RAS, Philippou H. The effect of blood coagulation factor XIII on fibrin clot structure and fibrinolysis. J Thromb Haemost 2014;12:197–205. 24261582 10.1111/jth.12455

[21] Collet JP , Park D, Lesty C, Soria J, Soria C, Montalescot G, Weisel JW. Influence of fibrin network conformation and fibrin fiber diameter on fibrinolysis speed: dynamic and structural approaches by confocal microscopy. Arterioscler Thromb Vasc Biol 2000;20:1354–1361. 10807754 10.1161/01.atv.20.5.1354

[22] Mullin JL , Gorkun OV, Lord ST. Decreased lateral aggregation of a variant recombinant fibrinogen provides insight into the polymerization mechanism. Biochemistry 2000;39:9843–9849. 10933802 10.1021/bi000045c

[23] Siudut J , Iwaniec T, Plens K, Pieters M, Undas A. Determinants of plasma fibrin clot lysis measured using three different assays in healthy subjects. Thromb Res 2021;197:1–7. 33157491 10.1016/j.thromres.2020.10.014

[24] Pieters M , Philippou H, Undas A, de Lange Z, Rijken DC, Mutch NJ, Subcommittee on Factor XIII and Fibrinogen, and the Subcommittee on Fibrinolysis . An international study on the feasibility of a standardized combined plasma clot turbidity and lysis assay: communication from the SSC of the ISTH. J Thromb Haemost 2018;16:1007–1012. 29658191 10.1111/jth.14002

[25] Pieters M , Undas A, Marchi R, de Maat MPM, Weisel JW, Ariëns RAS, Factor XIII and Fibrinogen Subcommittee of the Scientific Standardisation Committee of the International Society for Thrombosis and Haemostasis . An international study on the standardization of fibrin clot permeability measurement: methodological considerations and implications for healthy control values. J Thromb Haemost 2012;10:2179–2181. 23193585 10.1111/j.1538-7836.2012.04883.x

[26] He S , Cao H, Thålin C, Svensson J, Blombäck M, Wallén H. The clotting trigger is an important determinant for the coagulation pathway in vivo or in vitro-inference from data review. Semin Thromb Hemost 2021;47:63–73. 33348413 10.1055/s-0040-1718888

[27] Swanepoel AC , de Lange-Loots Z, Cockeran M, Pieters M. Lifestyle influences changes in fibrin clot properties over a 10-year period on a population level. Thromb Haemost 2022;122:67–79. 33906245 10.1055/a-1492-6143

[28] Whiting D , DiNardo JA. TEG And ROTEM: technology and clinical applications. Am J Hematol 2014;89:228–232. 24123050 10.1002/ajh.23599

[29] Hosokawa K , Ohnishi-Wada T, Nagasato T, Sameshima-Kaneko H, Oyamada C, Dahlen J. New methodological approaches for assessing thrombus formation in cardiovascular disease. Kardiol Pol 2020;78:667–673. 32633476 10.33963/KP.15493

[30] Lord ST . Fibrinogen and fibrin: scaffold proteins in hemostasis. Curr Opin Hematol 2007;14:236–241. 17414213 10.1097/MOH.0b013e3280dce58c

[31] Bagoly Z , Koncz Z, Hársfalvi J, Muszbek L. Factor XIII, clot structure, thrombosis. Thromb Res 2012;129:382–387. 22197181 10.1016/j.thromres.2011.11.040

[32] Stachowicz A , Zabczyk M, Natorska J, Suski M, Olszanecki R, Korbut R, Wiśniewski JR, Undas A. Differences in plasma fibrin clot composition in patients with thrombotic antiphospholipid syndrome compared with venous thromboembolism. Sci Rep 2018;8:17301. 30470809 10.1038/s41598-018-35034-x PMC6251889

[33] Bryk AH , Natorska J, Ząbczyk M, Zettl K, Wiśniewski JR, Undas A. Plasma fibrin clot proteomics in patients with acute pulmonary embolism: association with clot properties. J Proteomics 2020;229:103946. 32810596 10.1016/j.jprot.2020.103946

[34] Mann KG , Brummel K, Butenas S. What is all that thrombin for? J Thromb Haemost 2003;1:1504–1514. 12871286 10.1046/j.1538-7836.2003.00298.x

[35] Wolberg AS , Monroe DM, Roberts HR, Hoffman M. Elevated prothrombin results in clots with an altered fiber structure: a possible mechanism of the increased thrombotic risk. Blood 2003;101:3008–3013. 12506014 10.1182/blood-2002-08-2527

[36] Antovic A , Perneby C, Ekman GJ, Wallen HN, Hjemdahl P, Blombäck M, He S. Marked increase of fibrin gel permeability with very low dose ASA treatment. Thromb Res 2005;116:509–517. 16181986 10.1016/j.thromres.2005.02.007

[37] Ajjan RA , Standeven KF, Khanbhai M, Phoenix F, Gersh KC, Weisel JW, Kearney MT, Ariëns RAS, Grant PJ. Effects of aspirin on clot structure and fibrinolysis using a novel in vitro cellular system. Arterioscler Thromb Vasc Biol 2009;29:712–717. 19286636 10.1161/ATVBAHA.109.183707

[38] Undas A , Celinska-Löwenhoff M, Löwenhoff T, Szczeklik A. Statins, fenofibrate, and quinapril increase clot permeability and enhance fibrinolysis in patients with coronary artery disease. J Thromb Haemost 2006;4:1029–1036. 16689755 10.1111/j.1538-7836.2006.01882.x

[39] Undas A , Topór-Madry R, Tracz W. Simvastatin increases clot permeability and susceptibility to lysis in patients with LDL cholesterol below 3.4 mmol/l. Pol Arch Med Wewn 2009;119:354–359. 19694216

[40] Zolcinski M , Ciesla-Dul M, Undas A. Effects of atorvastatin on plasma fibrin clot properties in apparently healthy individuals and patients with previous venous thromboembolism. Thromb Haemost 2012;107:1180–1182. 22371154 10.1160/TH11-12-0851

[41] Siudut J , Ząbczyk M, Wołkow P, Polak M, Undas A, Jawień J. Intensive low-density lipoprotein cholesterol lowering improves fibrin clot properties: association with lipoproteins and C-reactive protein. Vascul Pharmacol 2022;144:106977. 35283275 10.1016/j.vph.2022.106977

[42] Rajzer M , Wojciechowska W, Kawecka-Jaszcz K, Undas A. Plasma fibrin clot properties in arterial hypertension and their modification by antihypertensive medication. Thromb Res 2012;130:99–103. 21917299 10.1016/j.thromres.2011.08.022

[43] Standeven KF , Ariëns RAS, Whitaker P, Ashcroft AE, Weisel JW, Grant PJ. The effect of dimethylbiguanide on thrombin activity, FXIII activation, fibrin polymerization, and fibrin clot formation. Diabetes 2002;51:189–197. 11756340 10.2337/diabetes.51.1.189

[44] Jörneskog G , Hansson L-O, Wallen NH, Yngen M, Blombäck M. Increased plasma fibrin gel porosity in patients with type I diabetes during continuous subcutaneous insulin infusion. J Thromb Haemost 2003;1:1195–1201. 12871319 10.1046/j.1538-7836.2003.00301.x

[45] Undas A , Brozek J, Jankowski M, Siudak Z, Szczeklik A, Jakubowski H. Plasma homocysteine affects fibrin clot permeability and resistance to lysis in human subjects. Arterioscler Thromb Vasc Biol 2006;26:1397–1404. 16574890 10.1161/01.ATV.0000219688.43572.75

[46] Gajos G , Zalewski J, Rostoff P, Nessler J, Piwowarska W, Undas A. Reduced thrombin formation and altered fibrin clot properties induced by polyunsaturated omega-3 fatty acids on top of dual antiplatelet therapy in patients undergoing percutaneous coronary intervention (OMEGA-PCI clot). Arterioscler Thromb Vasc Biol 2011;31:1696–1702. 21617138 10.1161/ATVBAHA.111.228593

[47] Ammollo CT , Semeraro F, Incampo F, Semeraro N, Colucci M. Dabigatran enhances clot susceptibility to fibrinolysis by mechanisms dependent on and independent of thrombin-activatable fibrinolysis inhibitor. J Thromb Haemost 2010;8:790–798. 20088944 10.1111/j.1538-7836.2010.03739.x

[48] Blombäck M , He S, Bark N, Wallen HN, Elg M. Effects on fibrin network porosity of anticoagulants with different modes of action and reversal by activated coagulation factor concentrate. Br J Haematol 2011;152:758–765. 21250974 10.1111/j.1365-2141.2010.08546.x

[49] Ząbczyk M , Majewski J, Karkowski G, Malinowski KP, Undas A. Vitamin K antagonists favourably modulate fibrin clot properties in patients with atrial fibrillation as early as after 3days of treatment: relation to coagulation factors and thrombin generation. Thromb Res 2015;136:832–838. 26319777 10.1016/j.thromres.2015.08.007

[50] Janion-Sadowska A , Natorska J, Siudut J, Ząbczyk M, Stanisz A, Undas A. Plasma fibrin clot properties in the G20210A prothrombin mutation carriers following venous thromboembolism: the effect of rivaroxaban. Thromb Haemost 2017;117:1739–1749. 28771277 10.1160/TH17-01-0060

[51] Carter RLR , Talbot K, Hur WS, Meixner SC, van der Gugten JG, Holmes DT, Côté HCF, Kastrup CJ, Smith TW, Lee AYY, Pryzdial ELG. Rivaroxaban and apixaban induce clotting factor Xa fibrinolytic activity. J Thromb Haemost 2018;16:2276–2288. 30176116 10.1111/jth.14281

[52] Ząbczyk M , Natorska J, Malinowski KP, Undas A. Effect of enoxaparin on plasma fibrin clot properties and fibrin structure in patients with acute pulmonary embolism. Vascul Pharmacol 2020;133–134:106783. 10.1016/j.vph.2020.106783 32835836

[53] Gauer JS , Riva N, Page EM, Philippou H, Makris M, Gatt A, Ariëns RAS. Effect of anticoagulants on fibrin clot structure: a comparison between vitamin K antagonists and factor Xa inhibitors. Res Pract Thromb Haemost 2020;4:1269–1281. 33313466 10.1002/rth2.12443 PMC7695561

[54] Sadowski M , Ząbczyk M, Undas A. Coronary thrombus composition: links with inflammation, platelet and endothelial markers. Atherosclerosis 2014;237:555–561. 25463088 10.1016/j.atherosclerosis.2014.10.020

[55] Gajos G , Siniarski A, Natorska J, Ząbczyk M, Siudut J, Malinowski KP, Gołębiowska-Wiatrak R, Rostoff P, Undas A. Polyhedrocytes in blood clots of type 2 diabetic patients with high cardiovascular risk: association with glycemia, oxidative stress and platelet activation. Cardiovasc Diabetol 2018;17:146. 30466424 10.1186/s12933-018-0789-6 PMC6251112

[56] Collet JP , Montalescot G, Lesty C, Weisel JW. A structural and dynamic investigation of the facilitating effect of glycoprotein IIb/IIIa inhibitors in dissolving platelet-rich clots. Circ Res 2002;90:428–434. 11884372 10.1161/hh0402.105095

[57] Williams S , Fatah K, Ivert T, Blombäck M. The effect of acetylsalicylic acid on fibrin gel lysis by tissue plasminogen activator. Blood Coagul Fibrinolysis 1995;6:718–725. 8825221 10.1097/00001721-199512000-00004

[58] Fatah K , Beving H, Albåge A, Ivert T, Blombäck M. Acetylsalicylic acid may protect the patient by increasing fibrin gel porosity. Is withdrawing of treatment harmful to the patient? Eur Heart J 1996;17:1362–1366. 8880021 10.1093/oxfordjournals.eurheartj.a015070

[59] Baker CJ , Smith SA, Morrissey JH. Polyphosphate in thrombosis, hemostasis, and inflammation. Res Pract Thromb Haemost 2019;3:18–25. 30656272 10.1002/rth2.12162 PMC6332810

[60] Mutch NJ , Engel R, de Willige SU, Philippou H, Ariëns RAS. Polyphosphate modifies the fibrin network and down-regulates fibrinolysis by attenuating binding of tPA and plasminogen to fibrin. Blood 2010;115:3980–3988. 20228273 10.1182/blood-2009-11-254029

[61] Cines DB , Lebedeva T, Nagaswami C, Hayes V, Massefski W, Litvinov RI, Rauova L, Lowery TJ, Weisel JW. Clot contraction: compression of erythrocytes into tightly packed polyhedra and redistribution of platelets and fibrin. Blood 2014;123:1596–1603. 24335500 10.1182/blood-2013-08-523860 PMC3945867

[62] Yang M , Huo X, Miao Z, Wang Y. Platelet glycoprotein IIb/IIIa receptor inhibitor tirofiban in acute ischemic stroke. Drugs 2019;79:515–529. 30838514 10.1007/s40265-019-01078-0

[63] Hantgan RR , Stahle MC, Jerome WG, Nagaswami C, Weisel JW. Tirofiban blocks platelet adhesion to fibrin with minimal perturbation of GpIIb/IIIa structure. Thromb Haemost 2002;87:910–917. 12038797

[64] Celińska-Lowenhoff M , Iwaniec T, Padjas A, Musiał J, Undas A. Altered fibrin clot structure/function in patients with antiphospholipid syndrome: association with thrombotic manifestation. Thromb Haemost 2014;112:287–296. 24652596 10.1160/TH13-11-0980

[65] Vikerfors A , Svenungsson E, Ågren A, Mobarrez F, Bremme K, Holmström M, Eelde A, Bruzelius M, Elgue G, Wallén H, Antovic A. Studies of fibrin formation and fibrinolytic function in patients with the antiphospholipid syndrome. Thromb Res 2014;133:936–944. 24630645 10.1016/j.thromres.2014.02.023

[66] Undas A , Kaczmarek P, Sladek K, Stepien E, Skucha W, Rzeszutko M, Gorkiewicz-Kot I, Tracz W. Fibrin clot properties are altered in patients with chronic obstructive pulmonary disease. Beneficial effects of simvastatin treatment. Thromb Haemost 2009;102:1176–1182. 19967149 10.1160/TH09-02-0118

[67] Kwasny-Krochin B , Gluszko P, Undas A. Unfavorably altered fibrin clot properties in patients with active rheumatoid arthritis. Thromb Res 2010;126:e11–e16. 20471669 10.1016/j.thromres.2010.04.007

[68] Pretorius E , Oberholzer HM, van der Spuy WJ, Swanepoel AC, Soma P. Scanning electron microscopy of fibrin networks in rheumatoid arthritis: a qualitative analysis. Rheumatol Int 2012;32:1611–1615. 21331577 10.1007/s00296-011-1805-2

[69] Bezuidenhout JA , Venter C, Roberts TJ, Tarr G, Kell DB, Pretorius E. Detection of citrullinated fibrin in plasma clots of rheumatoid arthritis patients and its relation to altered structural clot properties, disease-related inflammation and prothrombotic tendency. Front Immunol 2020;11:577523. 33424834 10.3389/fimmu.2020.577523 PMC7793985

[70] Undas A , Stepien E, Tracz W, Szczeklik A. Lipoprotein(a) as a modifier of fibrin clot permeability and susceptibility to lysis. J Thromb Haemost 2006;4:973–975. 16689745 10.1111/j.1538-7836.2006.01903.x

[71] Larsen JB , Aggerbeck MA, Larsen KM, Hvas CL, Hvas A-M. Fibrin network formation and lysis in septic shock patients. Int J Mol Sci 2021;22:9540. 34502446 10.3390/ijms22179540 PMC8431602

[72] Brubaker LS , Saini A, Nguyen TC, Martinez-Vargas M, Lam FW, Yao Q, Loor MM, Rosengart TK, Cruz MA. Aberrant fibrin clot structure visualized ex vivo in critically ill patients with severe acute respiratory syndrome coronavirus 2 infection. Crit Care Med 2022;50:e557–e568. 35170535 10.1097/CCM.0000000000005465 PMC9112654

[73] Wygrecka M , Birnhuber A, Seeliger B, Michalick L, Pak O, Schultz A-S, Schramm F, Zacharias M, Gorkiewicz G, David S, Welte T, Schmidt JJ, Weissmann N, Schermuly RT, Barreto G, Schaefer L, Markart P, Brack MC, Hippenstiel S, Kurth F, Sander LE, Witzenrath M, Kuebler WM, Kwapiszewska G, Preissner KT. Altered fibrin clot structure and dysregulated fibrinolysis contribute to thrombosis risk in severe COVID-19. Blood Adv 2022;6:1074–1087. 34861681 10.1182/bloodadvances.2021004816 PMC8648369

[74] Ekström M , Liska J, Eriksson P, Sverremark-Ekström E, Tornvall P. Stimulated in vivo synthesis of plasminogen activator inhibitor-1 in human adipose tissue. Thromb Haemost 2012;108:485–492. 22740034 10.1160/TH11-11-0822

[75] Longstaff C , Varjú I, Sótonyi P, Szabó L, Krumrey M, Hoell A, Bóta A, Varga Z, Komorowicz E, Kolev K. Mechanical stability and fibrinolytic resistance of clots containing fibrin, DNA, and histones. J Biol Chem 2013;288:6946–6956. 23293023 10.1074/jbc.M112.404301 PMC3591605

[76] Varjú I , Longstaff C, Szabó L, Farkas ÁZ, Varga-Szabó VJ, Tanka-Salamon A, Machovich R, Kolev K. DNA, histones and neutrophil extracellular traps exert anti-fibrinolytic effects in a plasma environment. Thromb Haemost 2015;113:1289–1298. 25789443 10.1160/TH14-08-0669

[77] Tóth E , Beinrohr L, Gubucz I, Szabó L, Tenekedjiev K, Nikolova N, Nagy AI, Hidi L, Sótonyi P, Szikora I, Merkely B, Kolev K. Fibrin to von Willebrand factor ratio in arterial thrombi is associated with plasma levels of inflammatory biomarkers and local abundance of extracellular DNA. Thromb Res 2022;209:8–15. 34844046 10.1016/j.thromres.2021.11.011

[78] de Boer OJ , Li X, Teeling P, Mackaay C, Ploegmakers HJ, van der Loos CM, Daemen MJ, de Winter RJ, van der Wal AC. Neutrophils, neutrophil extracellular traps and interleukin-17 associate with the organisation of thrombi in acute myocardial infarction. Thromb Haemost 2013;109:290–297. 23238559 10.1160/TH12-06-0425

[79] Ducroux C , di Meglio L, Loyau S, Delbosc S, Boisseau W, Deschildre C, Maacha MB, Blanc R, Redjem H, Ciccio G, Smajda S, Fahed R, Michel J-B, Piotin M, Salomon L, Mazighi M, Ho-Tin-Noe B, Desilles J-P. Thrombus neutrophil extracellular traps content impair tPA-induced thrombolysis in acute ischemic stroke. Stroke 2018;49:754–757. 29438080 10.1161/STROKEAHA.117.019896

[80] Farkas ÁZ , Farkas VJ, Gubucz I, Szabó L, Bálint K, Tenekedjiev K, Nagy AI, Sótonyi P, Hidi L, Nagy Z, Szikora I, Merkely B, Kolev K. Neutrophil extracellular traps in thrombi retrieved during interventional treatment of ischemic arterial diseases. Thromb Res 2019;175:46–52. 30703701 10.1016/j.thromres.2019.01.006

[81] Mołek P , Ząbczyk M, Malinowski KP, Natorska J, Undas A. Markers of NET formation and stroke risk in patients with atrial fibrillation: association with a prothrombotic state. Thromb Res 2022;213:1–7. 35276507 10.1016/j.thromres.2022.02.025

[82] Bryk AH , Prior SM, Plens K, Konieczynska M, Hohendorff J, Malecki MT, Butenas S, Undas A. Predictors of neutrophil extracellular traps markers in type 2 diabetes mellitus: associations with a prothrombotic state and hypofibrinolysis. Cardiovasc Diabetol 2019;18:49. 30992036 10.1186/s12933-019-0850-0 PMC6469138

[83] Casini A , Undas A, Palla R, Thachil J, de Moerloose P, Subcommittee on Factor XIII and Fibrinogen . Diagnosis and classification of congenital fibrinogen disorders: communication from the SSC of the ISTH. J Thromb Haemost 2018;16:1887–1890. 30076675 10.1111/jth.14216

[84] Marchi R , Lundberg U, Grimbergen J, Koopman J, Torres A, de Bosch NB, Haverkate F, Arocha Piñango CL. Fibrinogen Caracas V, an abnormal fibrinogen with an Aalpha 532 Ser–>Cys substitution associated with thrombosis. Thromb Haemost 2000;84:263–270. 10959699

[85] Koopman J , Haverkate F, Lord ST, Grimbergen J, Mannucci PM. Molecular basis of fibrinogen Naples associated with defective thrombin binding and thrombophilia. Homozygous substitution of B beta 68 Ala—Thr. J Clin Invest 1992;90:238–244. 1634610 10.1172/JCI115841 PMC443086

[86] Collet JP , Soria J, Mirshahi M, Hirsch M, Dagonnet FB, Caen J, Soria C. Dusart syndrome: a new concept of the relationship between fibrin clot architecture and fibrin clot degradability: hypofibrinolysis related to an abnormal clot structure. Blood 1993;82:2462–2469. 7691261

[87] Treliński J , Witkowski M, Chojnowski K, Neerman-Arbez M, Wypasek E, Undas A. Fibrinogen Łódź: a new cause of dysfibrinogenemia associated with recurrent thromboembolic arterial events. Pol Arch Intern Med 2019;129:934–935. 31596272 10.20452/pamw.15014

[88] Ariëns RAS . Novel mechanisms that regulate clot structure/function. Thromb Res 2016;141(Suppl. 2):S25–S27. 27207417 10.1016/S0049-3848(16)30358-9

[89] Allan P , Uitte de Willige S, Abou-Saleh RH, Connell SD, Ariëns RAS. Evidence that fibrinogen γ’ directly interferes with protofibril growth: implications for fibrin structure and clot stiffness. J Thromb Haemost 2012;10:1072–1080. 22463367 10.1111/j.1538-7836.2012.04717.x

[90] Domingues MM , Macrae FL, Duval C, McPherson HR, Bridge KI, Ajjan RA, Ridger VC, Connell SD, Philippou H, Ariëns RAS. Thrombin and fibrinogen γ’ impact clot structure by marked effects on intrafibrillar structure and protofibril packing. Blood 2016;127:487–495. 26608329 10.1182/blood-2015-06-652214

[91] Lovely RS , Falls LA, Al-Mondhiry HA, Chambers CE, Sexton GJ, Ni H, Farrell DH. Association of gammaA/gamma’ fibrinogen levels and coronary artery disease. Thromb Haemost 2002;88:26–31. 12152671

[92] Mannila MN , Lovely RS, Kazmierczak SC, Eriksson P, Samnegård A, Farrell DH, Hamsten A, Silveira A. Elevated plasma fibrinogen gamma’ concentration is associated with myocardial infarction: effects of variation in fibrinogen genes and environmental factors. J Thromb Haemost 2007;5:766–773. 17263791 10.1111/j.1538-7836.2007.02406.x

[93] Cheung EYL , Uitte de Willige S, Vos HL, Leebeek FWG, Dippel DWJ, Bertina RM, de Maat MPM. Fibrinogen gamma’ in ischemic stroke: a case-control study. Stroke 2008;39:1033–1035. 18239174 10.1161/STROKEAHA.107.495499

[94] Cheung EYL , Vos HL, Kruip MJHA, den Hertog HM, Jukema JW, de Maat MPM. Elevated fibrinogen gamma’ ratio is associated with cardiovascular diseases and acute phase reaction but not with clinical outcome. Blood 2009;114:4603–4604; author reply 4604–4605. 19965709 10.1182/blood-2009-08-236240

[95] Lovely RS , Yang Q, Massaro JM, Wang J, D’Agostino RB, O’Donnell CJ, Shannon J, Farrell DH. Assessment of genetic determinants of the association of γ’ fibrinogen in relation to cardiovascular disease. Arterioscler Thromb Vasc Biol 2011;31:2345–2352. 21757653 10.1161/ATVBAHA.111.232710 PMC3174319

[96] Pieters M , Kotze RC, Jerling JC, Kruger A, Ariëns RAS. Evidence that fibrinogen γ’ regulates plasma clot structure and lysis and relationship to cardiovascular risk factors in black Africans. Blood 2013;121:3254–3260. 23422752 10.1182/blood-2012-12-471482

[97] de Vries JJ , Snoek CJM, Rijken DC, de Maat MPM. Effects of post-translational modifications of fibrinogen on clot formation, clot structure, and fibrinolysis: a systematic review. Arterioscler Thromb Vasc Biol 2020;40:554–569. 31914791 10.1161/ATVBAHA.119.313626 PMC7043730

[98] Becatti M , Emmi G, Silvestri E, Bruschi G, Ciucciarelli L, Squatrito D, Vaglio A, Taddei N, Abbate R, Emmi L, Goldoni M, Fiorillo C, Prisco D. Neutrophil activation promotes fibrinogen oxidation and thrombus formation in Behçet disease. Circulation 2016;133:302–311. 26585672 10.1161/CIRCULATIONAHA.115.017738

[99] Undas A , Szułdrzynski K, Stepien E, Zalewski J, Godlewski J, Tracz W, Pasowicz M, Zmudka K. Reduced clot permeability and susceptibility to lysis in patients with acute coronary syndrome: effects of inflammation and oxidative stress. Atherosclerosis 2008;196:551–557. 17640649 10.1016/j.atherosclerosis.2007.05.028

[100] Becatti M , Marcucci R, Bruschi G, Taddei N, Bani D, Gori AM, Giusti B, Gensini GF, Abbate R, Fiorillo C. Oxidative modification of fibrinogen is associated with altered function and structure in the subacute phase of myocardial infarction. Arterioscler Thromb Vasc Biol 2014;34:1355–1361. 24790138 10.1161/ATVBAHA.114.303785

[101] White NJ , Wang Y, Fu X, Cardenas JC, Martin EJ, Brophy DF, Wade CE, Wang X, St John AE, Lim EB, Stern SA, Ward KR, López JA, Chung D. Post-translational oxidative modification of fibrinogen is associated with coagulopathy after traumatic injury. Free Radic Biol Med 2016;96:181–189. 27105953 10.1016/j.freeradbiomed.2016.04.023 PMC4912420

[102] Gołąb A , Plicner D, Rzucidło-Hymczak A, Tomkiewicz-Pająk L, Gawęda B, Kapelak B, Undas A. 8-Isoprostanes and asymmetric dimethylarginine as predictors of mortality in patients following coronary bypass surgery: a long-term follow-up study. J Clin Med 2022;11:246. 35011987 10.3390/jcm11010246 PMC8745691

[103] Mołek P , Chmiel J, Ząbczyk M, Malinowski KP, Natorska J, Undas A. Elevated 8-isoprostane concentration is associated with thromboembolic events in patients with atrial fibrillation. Int J Cardiol 2022;365:1–7. 35868355 10.1016/j.ijcard.2022.07.034

[104] Bryk-Wiązania AH , Cysewski D, Ocłoń E, Undas A. Mass-spectrometric identification of oxidative modifications in plasma-purified plasminogen: association with hypofibrinolysis in patients with acute pulmonary embolism. Biochem Biophys Res Commun 2022;621:53–58. 35810591 10.1016/j.bbrc.2022.06.063

[105] Pieters M , Covic N, Loots DT, van der Westhuizen FH, van Zyl DG, Rheeder P, Jerling JC, Weisel JW. The effect of glycaemic control on fibrin network structure of type 2 diabetic subjects. Thromb Haemost 2006;96:623–629. 17080220

[106] Dunn EJ , Ariëns RAS, Grant PJ. The influence of type 2 diabetes on fibrin structure and function. Diabetologia 2005;48:1198–1206. 15864538 10.1007/s00125-005-1742-2

[107] Pieters M , Covic N, van der Westhuizen FH, Nagaswami C, Baras Y, Toit Loots D, Jerling JC, Elgar D, Edmondson KS, van Zyl DG, Rheeder P, Weisel JW. Glycaemic control improves fibrin network characteristics in type 2 diabetes—a purified fibrinogen model. Thromb Haemost 2008;99:691–700. 18392327 10.1160/TH07-11-0699 PMC2854507

[108] Jakubowski H . Homocysteine modification in protein structure/function and human disease. Physiol Rev 2019;99:555–604. 30427275 10.1152/physrev.00003.2018

[109] McPherson HR , Duval C, Baker SR, Hindle MS, Cheah LT, Asquith NL, Domingues MM, Ridger VC, da Connell S, Naseem KM, Philippou H, Ajjan RA, Ariëns RA. Fibrinogen αC-subregions critically contribute blood clot fibre growth, mechanical stability, and resistance to fibrinolysis. Elife 2021;10:e68761. 34633287 10.7554/eLife.68761 PMC8553339

[110] Asquith NL , Duval C, Zhmurov A, Baker SR, McPherson HR, Domingues MM, Connell SDA, Barsegov V, Ariëns RAS. Fibrin protofibril packing and clot stability are enhanced by extended knob-hole interactions and catch-slip bonds. Blood Adv 2022;6:4015–4027. 35561308 10.1182/bloodadvances.2022006977 PMC9278297

[111] Duval C , Baranauskas A, Feller T, Ali M, Cheah LT, Yuldasheva NY, Baker SR, McPherson HR, Raslan Z, Bailey MA, Cubbon RM, Connell SD, Ajjan RA, Philippou H, Naseem KM, Ridger VC, Ariëns RAS. Elimination of fibrin γ-chain cross-linking by FXIIIa increases pulmonary embolism arising from murine inferior vena cava thrombi. Proc Natl Acad Sci U S A 2021;118:e2103226118. 10.1073/pnas.2103226118 PMC8271579 34183396

[112] Meltzer ME , Doggen CJM, de Groot PG, Rosendaal FR, Lisman T. Reduced plasma fibrinolytic capacity as a potential risk factor for a first myocardial infarction in young men. Br J Haematol 2009;145:121–127. 19170679 10.1111/j.1365-2141.2008.07569.x

[113] de Lange Z , Pieters M, Jerling JC, Kruger A, Rijken DC. Plasma clot lysis time and its association with cardiovascular risk factors in black Africans. PLoS One 2012;7:e48881. 23145007 10.1371/journal.pone.0048881 PMC3493613

[114] Fatah K , Silveira A, Tornvall P, Karpe F, Blombäck M, Hamsten A. Proneness to formation of tight and rigid fibrin gel structures in men with myocardial infarction at a young age. Thromb Haemost 1996;76:535–540. 8902992

[115] Carter AM , Cymbalista CM, Spector TD, Grant PJ, Investigators E. Heritability of clot formation, morphology, and lysis: the EuroCLOT study. Arterioscler Thromb Vasc Biol 2007;27:2783–2789. 17932316 10.1161/ATVBAHA.107.153221

[116] Eksteen P , Pieters M, de Lange Z, Kruger HS. The association of clot lysis time with total obesity is partly independent from the association of PAI-1 with central obesity in African adults. Thromb Res 2015;136:415–421. 26070447 10.1016/j.thromres.2015.05.033

[117] Mills JD , Ariëns RAS, Mansfield MW, Grant PJ. Altered fibrin clot structure in the healthy relatives of patients with premature coronary artery disease. Circulation 2002;106:1938–1942. 12370216 10.1161/01.cir.0000033221.73082.06

[118] Konieczynska M , Fil K, Bazanek M, Undas A. Prolonged duration of type 2 diabetes is associated with increased thrombin generation, prothrombotic fibrin clot phenotype and impaired fibrinolysis. Thromb Haemost 2014;111:685–693. 24306139 10.1160/TH13-07-0566

[119] Konieczyńska M , Bryk AH, Malinowski KP, Draga K, Undas A. Interplay between elevated cellular fibronectin and plasma fibrin clot properties in type 2 diabetes. Thromb Haemost 2017;117:1671–1678. 28569923 10.1160/TH17-04-0259

[120] Undas A , Topór-Madry R, Tracz W, Pasowicz M. Effect of cigarette smoking on plasma fibrin clot permeability and susceptibility to lysis. Thromb Haemost 2009;102:1289–1291. 19967167 10.1160/TH09-03-0187

[121] Barua RS , Sy F, Srikanth S, Huang G, Javed U, Buhari C, Margosan D, Ambrose JA. Effects of cigarette smoke exposure on clot dynamics and fibrin structure: an ex vivo investigation. Arterioscler Thromb Vasc Biol 2010;30:75–79. 19815816 10.1161/ATVBAHA.109.195024

[122] Pieters M , Guthold M, Nunes CM, de Lange Z. Interpretation and validation of maximum absorbance data obtained from turbidimetry analysis of plasma clots. Thromb Haemost 2020;120:44–54. 31752041 10.1055/s-0039-1698460 PMC7369642

[123] Pieters M , Vorster HH, Jerling JC, Venter CS, Kotze RCM, Bornman E, Malfliet JJMC, Rijken DC. The effect of ethanol and its metabolism on fibrinolysis. Thromb Haemost 2010;104:724–733. 20664891 10.1160/TH10-01-0048

[124] Siudut J , Natorska J, Wypasek E, Wiewiórka Ł, Ostrowska-Kaim E, Wiśniowska-Śmiałek S, Plens K, Musialek P, Legutko J, Undas A. Apolipoproteins and lipoprotein(a) as factors modulating fibrin clot properties in patients with severe aortic stenosis. Atherosclerosis 2022;344:49–56. 35134656 10.1016/j.atherosclerosis.2022.01.011

[125] Guimarães AHC , de Bruijne ELE, Lisman T, Dippel DWJ, Deckers JW, Poldermans D, Rijken DC, Leebeek FWG. Hypofibrinolysis is a risk factor for arterial thrombosis at young age. Br J Haematol 2009;145:115–120. 19183334 10.1111/j.1365-2141.2008.07568.x

[126] Shimomura I , Funahashi T, Takahashi M, Maeda K, Kotani K, Nakamura T, Yamashita S, Miura M, Fukuda Y, Takemura K, Tokunaga K, Matsuzawa Y. Enhanced expression of PAI-1 in visceral fat: possible contributor to vascular disease in obesity. Nat Med 1996;2:800–803. 8673927 10.1038/nm0796-800

[127] Bryk-Wiązania AH , Undas A. Hypofibrinolysis in type 2 diabetes and its clinical implications: from mechanisms to pharmacological modulation. Cardiovasc Diabetol 2021;20:191. 34551784 10.1186/s12933-021-01372-w PMC8459566

[128] Dunn EJ , Philippou H, Ariëns RAS, Grant PJ. Molecular mechanisms involved in the resistance of fibrin to clot lysis by plasmin in subjects with type 2 diabetes mellitus. Diabetologia 2006;49:1071–1080. 16538489 10.1007/s00125-006-0197-4

[129] Hess K , Alzahrani SH, Mathai M, Schroeder V, Carter AM, Howell G, Koko T, Strachan MWJ, Price JF, Smith KA, Grant PJ, Ajjan RA. A novel mechanism for hypofibrinolysis in diabetes: the role of complement C3. Diabetologia 2012;55:1103–1113. 21918806 10.1007/s00125-011-2301-7

[130] King R , Tiede C, Simmons K, Fishwick C, Tomlinson D, Ajjan R. Inhibition of complement C3 and fibrinogen interaction: a potential novel therapeutic target to reduce cardiovascular disease in diabetes. Lancet 2015;385(Suppl. 1):S57. 10.1016/S0140-6736(15)60372-5 26312879

[131] Bryk AH , Konieczyńska M, Polak M, Plicner D, Bochenek M, Undas A. Plasma fibrin clot properties and cardiovascular mortality in patients with type 2 diabetes: a long-term follow-up study. Cardiovasc Diabetol 2021;20:47. 33602240 10.1186/s12933-021-01230-9 PMC7893920

[132] Chow E , Iqbal A, Walkinshaw E, Phoenix F, Macdonald IA, Storey RF, Ajjan R, Heller SR. Prolonged prothrombotic effects of antecedent hypoglycemia in individuals with type 2 diabetes. Diabetes Care 2018;41:2625–2633. 30327358 10.2337/dc18-0050

[133] Collet JP , Allali Y, Lesty C, Tanguy ML, Silvain J, Ankri A, Blanchet B, Dumaine R, Gianetti J, Payot L, Weisel JW, Montalescot G. Altered fibrin architecture is associated with hypofibrinolysis and premature coronary atherothrombosis. Arterioscler Thromb Vasc Biol 2006;26:2567–2573. 16917107 10.1161/01.ATV.0000241589.52950.4c

[134] Boffa MB , Koschinsky ML. Oxidized phospholipids as a unifying theory for lipoprotein(a) and cardiovascular disease. Nat Rev Cardiol 2019;16:305–318. 30675027 10.1038/s41569-018-0153-2

[135] Boffa MB . Beyond fibrinolysis: the confounding role of Lp(a) in thrombosis. Atherosclerosis 2022;349:72–81. 35606079 10.1016/j.atherosclerosis.2022.04.009

[136] Dobrosielski DA . How can exercise reduce cardiovascular disease risk? A primer for the clinician. Pol Arch Intern Med 2021;131:16122. 34706491 10.20452/pamw.16122

[137] Davies NA , Llwyd O, Brugniaux JV, Davies GR, Marley CJ, Hodson D, Lawrence MJ, D’Silva LA, Morris RHK, Hawkins K, Williams PR, Bailey DM, Evans PA. Effects of exercise intensity on clot microstructure and mechanical properties in healthy individuals. Thromb Res 2016;143:130–136. 27240111 10.1016/j.thromres.2016.05.018

[138] Borissoff JI , Spronk HMH, ten Cate H. The hemostatic system as a modulator of atherosclerosis. N Engl J Med 2011;364:1746–1760. 21542745 10.1056/NEJMra1011670

[139] Kietsiriroje N , Ariëns RAS, Ajjan RA. Fibrinolysis in acute and chronic cardiovascular disease. Semin Thromb Hemost 2021;47:490–505. 33878782 10.1055/s-0040-1718923

[140] Sumaya W , Wallentin L, James SK, Siegbahn A, Gabrysch K, Himmelmann A, Ajjan RA, Storey RF. Impaired fibrinolysis predicts adverse outcome in acute coronary syndrome patients with diabetes: a PLATO sub-study. Thromb Haemost 2020;120:412–422. 31975352 10.1055/s-0039-1701011 PMC7286125

[141] Leander K , Blombäck M, Wallén H, He S. Impaired fibrinolytic capacity and increased fibrin formation associate with myocardial infarction. Thromb Haemost 2012;107:1092–1099. 22476576 10.1160/TH11-11-0760

[142] Zalewski J , Bogaert J, Sadowski M, Woznicka O, Doulaptsis K, Ntoumpanaki M, Ząbczyk M, Nessler J, Undas A. Plasma fibrin clot phenotype independently affects intracoronary thrombus ultrastructure in patients with acute myocardial infarction. Thromb Haemost 2015;113:1258–1269. 25739375 10.1160/TH14-09-0801

[143] Ząbczyk M , Natorska J, Zalewski J, Undas A. Fibrin biofilm can be detected on intracoronary thrombi aspirated from patients with acute myocardial infarction. Cardiovasc Res 2019;115:1026–1028. 30689756 10.1093/cvr/cvz019

[144] Macrae FL , Duval C, Papareddy P, Baker SR, Yuldasheva N, Kearney KJ, McPherson HR, Asquith N, Konings J, Casini A, Degen JL, Connell SD, Philippou H, Wolberg AS, Herwald H, Ariëns RA. A fibrin biofilm covers blood clots and protects from microbial invasion. J Clin Invest 2018;128:3356–3368. 29723163 10.1172/JCI98734 PMC6063501

[145] Ząbczyk M , Sadowski M, Zalewski J, Undas A. Polyhedrocytes in intracoronary thrombi from patients with ST-elevation myocardial infarction. Int J Cardiol 2015;179:186–187. 25464440 10.1016/j.ijcard.2014.10.004

[146] Chernysh IN , Nagaswami C, Kosolapova S, Peshkova AD, Cuker A, Cines DB, Cambor CL, Litvinov RI, Weisel JW. The distinctive structure and composition of arterial and venous thrombi and pulmonary emboli. Sci Rep 2020;10:5112. 32198356 10.1038/s41598-020-59526-x PMC7083848

[147] Tutwiler V , Peshkova AD, Andrianova IA, Khasanova DR, Weisel JW, Litvinov RI. Contraction of blood clots is impaired in acute ischemic stroke. Arterioscler Thromb Vasc Biol 2017;37:271–279. 27908894 10.1161/ATVBAHA.116.308622 PMC5269459

[148] Zabczyk M , Natorska J, Undas A. Erythrocyte compression index is impaired in patients with residual vein obstruction. J Thromb Thrombolysis 2018;46:31–38. 29589187 10.1007/s11239-018-1650-1 PMC5994218

[149] Gersh KC , Nagaswami C, Weisel JW. Fibrin network structure and clot mechanical properties are altered by incorporation of erythrocytes. Thromb Haemost 2009;102:1169–1175. 19967148 10.1160/TH09-03-0199 PMC2840711

[150] Guedes AF , Carvalho FA, Domingues MM, Macrae FL, McPherson HR, Santos NC, Ariёns RAS. Sensing adhesion forces between erythrocytes and γ’ fibrinogen, modulating fibrin clot architecture and function. Nanomedicine 2018;14:909–918. 29410160 10.1016/j.nano.2018.01.006

[151] Siniarski A , Baker SR, Duval C, Malinowski KP, Gajos G, Nessler J, Ariëns RAS. Quantitative analysis of clot density, fibrin fiber radius, and protofibril packing in acute phase myocardial infarction. Thromb Res 2021;205:110–119. 34298252 10.1016/j.thromres.2021.06.024

[152] Dunn EJ , Ariëns RA, de Lange M, Snieder H, Turney JH, Spector TD, Grant PJ. Genetics of fibrin clot structure: a twin study. Blood 2004;103:1735–1740. 14604965 10.1182/blood-2003-07-2247

[153] Reddel CJ , Curnow JL, Voitl J, Rosenov A, Pennings GJ, Morel-Kopp M-C, Brieger DB. Detection of hypofibrinolysis in stable coronary artery disease using the overall haemostatic potential assay. Thromb Res 2013;131:457–462. 23582780 10.1016/j.thromres.2013.03.015

[154] Ramanathan R , Gram JB, Sidelmann JJ, Dey D, Kusk MW, Nørgaard BL, Sand NPR. Sex difference in fibrin clot lysability: association with coronary plaque composition. Thromb Res 2019;174:129–136. 30597343 10.1016/j.thromres.2018.12.020

[155] Neergaard-Petersen S , Larsen SB, Grove EL, Kristensen SD, Ajjan RA, Hvas A-M. Imbalance between fibrin clot formation and fibrinolysis predicts cardiovascular events in patients with stable coronary artery disease. Thromb Haemost 2020;120:75–82. 31733633 10.1055/s-0039-1700873

[156] Siegerink B , Meltzer ME, de Groot PG, Algra A, Lisman T, Rosendaal FR. Clot lysis time and the risk of myocardial infarction and ischaemic stroke in young women; results from the RATIO case-control study. Br J Haematol 2012;156:252–258. 22082241 10.1111/j.1365-2141.2011.08935.x

[157] Gołąb A , Plicner D, Wypasek E, Natorska J, Kapelak B, Plens K, Undas A. Impaired fibrin clot lysis is associated with increased mortality in patients after coronary artery bypass grafting: a long-term follow-up study. Eur J Clin Invest 2022;52:e13775. 35313018 10.1111/eci.13775

[158] Neergaard-Petersen S , Hvas A-M, Kristensen SD, Grove EL, Larsen SB, Phoenix F, Kurdee Z, Grant PJ, Ajjan RA. The influence of type 2 diabetes on fibrin clot properties in patients with coronary artery disease. Thromb Haemost 2014;112:1142–1150. 25187394 10.1160/TH14-05-0468

[159] Bochenek M , Zalewski J, Sadowski J, Undas A. Type 2 diabetes as a modifier of fibrin clot properties in patients with coronary artery disease. J Thromb Thrombolysis 2013;35:264–270. 23086579 10.1007/s11239-012-0821-8 PMC3549239

[160] Winther-Larsen A , Christiansen MK, Larsen SB, Nyegaard M, Neergaard-Petersen S, Ajjan RA, Würtz M, Grove EL, Jensen HK, Kristensen SD, Hvas A-M. The ABO locus is associated with increased fibrin network formation in patients with stable coronary artery disease. Thromb Haemost 2020;120:1248–1256. 32604426 10.1055/s-0040-1713753

[161] Ząbczyk M , Natorska J, Undas A. Fibrin clot properties in atherosclerotic vascular disease: from pathophysiology to clinical outcomes. J Clin Med 2021;10:2999. 34279484 10.3390/jcm10132999 PMC8268932

[162] Palka I , Nessler J, Nessler B, Piwowarska W, Tracz W, Undas A. Altered fibrin clot properties in patients with chronic heart failure and sinus rhythm: a novel prothrombotic mechanism. Heart 2010;96:1114–1118. 20610458 10.1136/hrt.2010.192740

[163] Lau YC , Xiong Q, Ranjit P, Lip GYH, Blann AD. Laboratory assessment of anti-thrombotic therapy in heart failure, atrial fibrillation and coronary artery disease: insights using thrombelastography and a micro-titre plate assay of thrombogenesis and fibrinolysis. J Thromb Thrombolysis 2016;42:233–244. 26942726 10.1007/s11239-016-1344-5 PMC4912975

[164] Undas A , Slowik A, Wolkow P, Szczudlik A, Tracz W. Fibrin clot properties in acute ischemic stroke: relation to neurological deficit. Thromb Res 2010;125:357–361. 19942259 10.1016/j.thromres.2009.11.013

[165] Bembenek JP , Niewada M, Siudut J, Plens K, Członkowska A, Undas A. Fibrin clot characteristics in acute ischaemic stroke patients treated with thrombolysis: the impact on clinical outcome. Thromb Haemost 2017;117:1440–1447. 28382369 10.1160/TH16-12-0954

[166] Jolugbo P , Ariëns RAS. Thrombus composition and efficacy of thrombolysis and thrombectomy in acute ischemic stroke. Stroke 2021;52:1131–1142. 33563020 10.1161/STROKEAHA.120.032810 PMC7610448

[167] Anzej S , Bozic M, Antovic A, Peternel P, Gaspersic N, Rot U, Tratar G, Stegnar M. Evidence of hypercoagulability and inflammation in young patients long after acute cerebral ischaemia. Thromb Res 2007;120:39–46. 17034835 10.1016/j.thromres.2006.08.005

[168] Undas A , Podolec P, Zawilska K, Pieculewicz M, Jedliński I, Stepień E, Konarska-Kuszewska E, Weglarz P, Duszyńska M, Hanschke E, Przewlocki T, Tracz W. Altered fibrin clot structure/function in patients with cryptogenic ischemic stroke. Stroke 2009;40:1499–1501. 19246700 10.1161/STROKEAHA.108.532812

[169] Bhasin N , Ariëns RAS, West RM, Parry DJ, Grant PJ, Scott DJA. Altered fibrin clot structure and function in the healthy first-degree relatives of subjects with intermittent claudication. J Vasc Surg 2008;48:1497–1503.e1. 18829228 10.1016/j.jvs.2008.07.010

[170] Bhasin N , Parry DJ, Scott DJA, Ariëns RAS, Grant PJ, West RM. Regarding ‘altered fibrin clot structure and function in individuals with intermittent claudication’. J Vasc Surg 2009;49:1088–1089. 19147322 10.1016/j.jvs.2008.11.028

[171] Undas A , Nowakowski T, Cieśla-Dul M, Sadowski J. Abnormal plasma fibrin clot characteristics are associated with worse clinical outcome in patients with peripheral arterial disease and thromboangiitis obliterans. Atherosclerosis 2011;215:481–486. 21324459 10.1016/j.atherosclerosis.2010.12.040

[172] Karpińska IA , Nowakowski T, Wypasek E, Plens K, Undas A. A prothrombotic state and denser clot formation in patients following acute limb ischemia of unknown cause. Thromb Res 2020;187:32–38. 31951936 10.1016/j.thromres.2020.01.008

[173] Okraska-Bylica A , Wilkosz T, Słowik L, Bazanek M, Konieczyńska M, Undas A. Altered fibrin clot properties in patients with premature peripheral artery disease. Pol Arch Med Wewn 2012;122:608–615. 23235487 10.20452/pamw.1535

[174] Nowakowski T , Malinowski KP, Niżankowski R, Iwaniec T, Undas A. Restenosis is associated with prothrombotic plasma fibrin clot characteristics in endovascularly treated patients with critical limb ischemia. J Thromb Thrombolysis 2019;47:540–549. 30762155 10.1007/s11239-019-01826-9 PMC6476846

[175] Boriani G , Valenti AC, Vitolo M. Biomarkers in atrial fibrillation: a constant search for simplicity, practicality, and cost-effectiveness. Kardiol Pol 2021;79:243–245. 33779121 10.33963/KP.15889

[176] Matusik PT , Leśniak WJ, Heleniak Z, Undas A. Thromboembolism and bleeding in patients with atrial fibrillation and stage 4 chronic kidney disease: impact of biomarkers. Kardiol Pol 2021;79:1086–1092. 34392517 10.33963/KP.a2021.0088

[177] Krittayaphong R , Methavigul K. Biomarkers for atrial fibrillation and chronic kidney disease: what is the evidence? Kardiol Pol 2021;79:1058–1059. 34611881 10.33963/KP.a2021.0124

[178] Undas A , Drabik L, Potpara T. Bleeding in anticoagulated patients with atrial fibrillation: practical considerations. Kardiol Pol 2020;78:105–116. 32108755 10.33963/KP.15205

[179] Drabik L , Wołkow P, Undas A. Denser plasma clot formation and impaired fibrinolysis in paroxysmal and persistent atrial fibrillation while on sinus rhythm: association with thrombin generation, endothelial injury and platelet activation. Thromb Res 2015;136:408–414. 26048399 10.1016/j.thromres.2015.05.028

[180] Matusik PT , Matusik PS, Kornacewicz-Jach Z, Małecka B, Ząbek A, Undas A. Elevated NT-proBNP is associated with unfavorably altered plasma fibrin clot properties in atrial fibrillation. Int J Cardiol 2017;243:244–250. 28571619 10.1016/j.ijcard.2017.05.060

[181] Drabik L , Wołkow P, Undas A. Fibrin clot permeability as a predictor of stroke and bleeding in anticoagulated patients with atrial fibrillation. Stroke 2017;48:2716–2722. 28904234 10.1161/STROKEAHA.117.018143

[182] Janion-Sadowska A , Chrapek M, Konieczyńska M, Undas A. Altered fibrin clot properties predict stroke and bleedings in patients with atrial fibrillation on rivaroxaban. *Stroke*; doi:10.1161/STROKEAHA.118.023712. Published online ahead of print 21 November 2018. 30580709

[183] Scott DJA , Prasad P, Philippou H, Rashid ST, Sohrabi S, Whalley D, Kordowicz A, Tang Q, West RM, Johnson A, Woods J, Ajjan RA, Ariëns RAS. Clot architecture is altered in abdominal aortic aneurysms and correlates with aneurysm size. Arterioscler Thromb Vasc Biol 2011;31:3004–3010. 21921257 10.1161/ATVBAHA.111.236786

[184] Sundermann AC , Saum K, Conrad KA, Russell HM, Edwards TL, Mani K, Björck M, Wanhainen A, Owens AP. Prognostic value of D-dimer and markers of coagulation for stratification of abdominal aortic aneurysm growth. Blood Adv 2018;2:3088–3096. 30442686 10.1182/bloodadvances.2017013359 PMC6258923

[185] Hołda MK , Iwaszczuk P, Wszołek K, Chmiel J, Brzychczy A, Trystuła M, Misztal M. Coexistence and management of abdominal aortic aneurysm and coronary artery disease. Cardiol J 2020;27:384–393. 30234902 10.5603/CJ.a2018.0101 PMC8016013

[186] Natorska J , Wypasek E, Grudzień G, Sadowski J, Undas A. Impaired fibrinolysis is associated with the severity of aortic stenosis in humans. J Thromb Haemost 2013;11:733–740. 23289423 10.1111/jth.12122

[187] Mazur P , Myć J, Natorska J, Plens K, Plicner D, Grudzień G, Kapelak B, Undas A. Impaired fibrinolysis in degenerative mitral and aortic valve stenosis. J Thromb Thrombolysis 2018;46:193–202. 29855781 10.1007/s11239-018-1687-1

[188] Siudut J , Natorska J, Wypasek E, Wiewiórka Ł, Ostrowska-Kaim E, Wiśniowska-Śmiałek S, Plens K, Legutko J, Undas A. Impaired fibrinolysis in patients with isolated aortic stenosis is associated with enhanced oxidative stress. J Clin Med 2020;9:2002. 32630544 10.3390/jcm9062002 PMC7355626

[189] Jaworska-Wilczyńska M , Natorska J, Siudut J, Marzec K, Kowalik I, Hryniewiecki T, Undas A. Patients scheduled for TAVI tend to form abnormal fibrin clots more resistant to lysis: the impact of age. Kardiol Pol;79:796–803. 34002842 10.33963/KP.a2021.0005

[190] Natorska J . Diabetes mellitus as a risk factor for aortic stenosis: from new mechanisms to clinical implications. Kardiol Pol 2021;79:1060–1067. 34643267 10.33963/KP.a2021.0137

